# Cerebellar transcranial magnetic stimulation in psychotic disorders: intermittent, continuous, and sham theta-burst stimulation on time perception and symptom severity

**DOI:** 10.3389/fpsyt.2023.1218321

**Published:** 2023-11-13

**Authors:** Ann K. Shinn, Aura M. Hurtado-Puerto, Youkyung S. Roh, Victoria Ho, Melissa Hwang, Bruce M. Cohen, Dost Öngür, Joan A. Camprodon

**Affiliations:** ^1^Psychotic Disorders Division, McLean Hospital, Belmont, MA, United States; ^2^Department of Psychiatry, Harvard Medical School, Boston, MA, United States; ^3^Laboratory for Neuropsychiatry and Neuromodulation, Massachusetts General Hospital, Boston, MA, United States; ^4^Program for Neuropsychiatric Research, McLean Hospital, Belmont, MA, United States

**Keywords:** cerebellum, neuromodulation, schizophrenia, bipolar disorder, interval discrimination task

## Abstract

**Background:**

The cerebellum contributes to the precise timing of non-motor and motor functions, and cerebellum abnormalities have been implicated in psychosis pathophysiology. In this study, we explored the effects of cerebellar theta burst stimulation (TBS), an efficient transcranial magnetic stimulation protocol, on temporal discrimination and self-reported mood and psychotic symptoms.

**Methods:**

We conducted a case-crossover study in which patients with psychosis (schizophrenias, schizoaffective disorders, or bipolar disorders with psychotic features) were assigned to three sessions of TBS to the cerebellar vermis: one session each of intermittent (iTBS), continuous (cTBS), and sham TBS. Of 28 enrolled patients, 26 underwent at least one TBS session, and 20 completed all three. Before and immediately following TBS, participants rated their mood and psychotic symptoms and performed a time interval discrimination task (IDT). We hypothesized that cerebellar iTBS and cTBS would modulate these measures in opposing directions, with iTBS being adaptive and cTBS maladaptive.

**Results:**

Reaction time (RT) in the IDT decreased significantly after iTBS vs. Sham (LS-mean difference = −73.3, *p* = 0.0001, Cohen’s *d* = 1.62), after iTBS vs. cTBS (LS-mean difference = −137.6, *p* < 0.0001, *d* = 2.03), and after Sham vs. cTBS (LS-mean difference = −64.4, *p* < 0.0001, *d* = 1.33). We found no effect on IDT accuracy. We did not observe any effects on symptom severity after correcting for multiple comparisons.

**Conclusion:**

We observed a frequency-dependent dissociation between the effects of iTBS vs. cTBS to the cerebellar midline on the reaction time of interval discrimination in patients with psychosis. iTBS showed improved (adaptive) while cTBS led to worsening (maladaptive) speed of response. These results demonstrate behavioral target engagement in a cognitive dimension of relevance to patients with psychosis and generate testable hypotheses about the potential therapeutic role of cerebellar iTBS in this clinical population.

**Clinical Trial Registration:**

clinicaltrials.gov, identifier NCT02642029.

## 1. Introduction

Psychotic disorders such as schizophrenias (SZ), schizoaffective disorders (SZA), and psychotic bipolar disorders (BD) are severe illnesses that involve disturbances in multiple domains (e.g., thought, behavior, language, cognition, perception, and mood). Despite significant efforts to identify what causes these conditions, a unified understanding of the pathophysiology underlying SZ and psychotic disorders remains elusive. The past few decades have seen increasing interest in the potential role of the cerebellum in disorders of cognition, behavior, and affect (1–4), and a growing literature provides evidence of cerebellar abnormalities in both SZ and BD supporting its role in the pathophysiology of psychosis (5–9). Though the cerebellum was traditionally thought to be involved solely in the homeostatic control of motor activities, it is now well established that the cerebellum is reciprocally connected to multimodal association areas (10–19) in addition to motor cortex, and that it serves a domain-general role in processing and coordinating diverse inputs (20–25).

The notion that the cerebellum applies a universal computation to diverse inputs offers an appealing and potentially unifying framework by which to explain the myriad symptoms in psychotic disorders. One of the proposed mechanisms is that the cerebellum performs a multidomain temporal coordination across tasks and brain functions (26–29). Keele and Ivry conceptualized the cerebellum as an “internal clock” that performs temporal computations in both the motor and non-motor domains, hypothesizing that the cerebellum’s highly regular cellular organization allows it to produce and coordinate precise temporal delays (26). Indeed, a vast literature corroborates the importance of the cerebellum in timing operations (30). While the cerebellum is not the sole brain area involved in temporal processing (31), it possesses intrinsic timing mechanisms that are not dependent on any network-generated time-varying input (32–36), and is particularly critical for timing functions requiring sub-second precision (37–39).

Precise timing is critical for synchronizing and coordinating diverse tasks. Cerebellar timing functions might play a role as a cognitive and “emotional pacemaker” (40), which, if disrupted, may result in incoordination, or “dysmetria,” of cognitive, behavioral, affective, and perceptual processes. Such dysmetria, in turn, may result in symptoms of psychosis (1–4). Consistent with this idea, impairments in time perception have been observed in both SZ and BD. Experimental methods commonly used to investigate time perception (i.e., processes related to the explicit judgment of the duration of events or the production of time intervals) (41) include verbal estimation of intervals (in which participants are presented with a time interval and instructed to estimate the interval duration in seconds or minutes), the repetitive finger tapping task (in which participants tap in time with computer-generated tones, then try to tap at the same pace after the tones are discontinued), the interval discrimination task (in which participants compare the duration of an experimental interval with a standard duration), and the temporal bisection task (in which participants judge whether a stimulus is most similar to a long or short anchor interval) (41, 42). Compared to healthy individuals, people with SZ are less accurate in estimating time durations across a wide range of timing tasks and independent of the duration of intervals that have been tested, suggesting that people with SZ have a primary timing deficit [see meta-analysis (42)]. Studies also indicate that time perception in SZ compared to healthy individuals is more variable [see meta-analysis (41)]. Interestingly, a functional neuroimaging study showed that timing deficits in schizophrenia were associated with alterations in the cerebellum, basal ganglia, supplementary motor area (SMA), and insula, among other brain areas (43). Critically, in this study, time processing deficits were associated with hyperactivation in the cerebellar hemispheres but hypoactivation in the cerebellar vermis (43).

Though the literature on timing abnormalities in BD is more sparse, BD patients are reported to have increased timing variability, as measured by the finger tapping task (44) and the temporal bisection task (45, 46). Notably, one of the latter studies investigated time perception in both SZ and BD and found that the bisection point did not differ across the patient groups, suggesting that both SZ spectrum disorders and BD are associated with disruptions in internal timing mechanisms.

While a growing body of research has contributed to the characterization of timing deficits in psychotic disorders, it remains unclear if such deficits in time perception can be improved. Parker et al. provided evidence, in rodents, of a relationship between timing and fronto-cerebellar circuitry by directly manipulating activity at the cerebellum (47). The authors showed that pharmacological inactivation of either lateral cerebellar nuclei (LCN) or medial frontal cortex (MFC) led to impaired performance by rodents on an interval timing task, and that delta-frequency optogenetic stimulation of the LCN in MFC-inactivated rodents rescued both behavioral timing deficits and MFC activity. Using the human version of the timing task, this group also found impaired interval timing and attenuated MFC delta activity in SZ relative to healthy participants (47). Though the patient data provide parallels with the rodent model and are highly suggestive, the human study was observational, involving no experimental interventions, and hence was limited in its capacity to infer causality.

Transcranial magnetic stimulation (TMS) is a noninvasive method of neuromodulation in which magnetic fields applied over the scalp induce electrical currents to excite or inhibit specific regions of the underlying neural tissue and transynaptically modulate the connectivity of those regions with distal nodes within a given functional network (48). The ability of TMS to up-or down-regulate brain regions and networks has been leveraged to study the functional significance of brain regions and circuits, relying on its interventional nature to establish *causal* relationships between brain physiology and behavior (49). Theta burst stimulation (TBS), a TMS protocol that in its most common variation administers bursts of three 50 Hz pulses (in the gamma range) every 200 ms (i.e., 5 Hz, in the theta range), induces longer lasting neuroplastic effects despite the much shorter stimulation times compared with traditional repetitive TMS (e.g., in the 1–20 Hz range) (50, 51). In the primary motor cortex, where the effects of TBS have been most investigated, the two most common TBS protocols— continuous TBS (cTBS), whereby TBS is given continuously, and intermittent TBS (iTBS), in which a 2 s train of TBS is repeated every 10s with an inter-train interval pause of 8 s— have opposing effects on cortical excitability (51): cTBS produces a predominantly long-term depression (LTD)-like inhibitory effect that reduces the amplitude of motor evoked potentials (MEP), while iTBS has an overall long-term potentiation (LTP)-like facilitatory effect, enhancing MEP amplitudes (50, 51) (we do not describe these effects fully as LTD and LTP as these are synaptic physiology phenomena and TBS engages populations of neurons at a larger scale than individual synapses). It is unclear if the TBS parameters that alter cortical excitability in the motor cortex produce the same effects in the cerebellum, which has a distinctive architecture consisting of cell types (e.g., granule cells and Purkinje cells) that are unique to the cerebellum and in a histological configuration quite different from the 6-layer organization of the primary motor cortex. Nevertheless, TBS has been safely administered to the cerebellum in >60 studies to date, ranging from those in patients with neuropsychiatric conditions to investigations of either motor or non-motor functions in healthy individuals [see review (52)].

Previous cerebellar TMS studies in SZ have uncovered diverse effects of cerebellar stimulation on cognition and symptoms, especially negative symptoms (53–57) [though also see (58, 59) for negative studies]. The study by Brady et al., which found that cerebellar TMS (iTBS) not only improved negative symptoms but also restored associated dorsolateral prefrontal-cerebellar resting state circuit abnormalities (56), additionally provides insights into the neural circuitry underlying negative symptoms. Similarly, Tikka et al.’s finding that reductions in resting state gamma power in left frontal and left temporal regions accompanied reductions in negative and depressive symptoms after cerebellar 5–7 Hz TMS (54) provides clues about potential mechanisms by which cerebellar stimulation may result in symptom improvement.

Notably, the participants in the studies published to date received only putatively excitatory TMS. Investigating both excitatory and inhibitory TMS has the potential to provide additional causal mechanistic insights and offers a non-invasive study design in humans that parallels the experimental interventions to the cerebellum performed by Parker et al. in rodents combining pharmacological inactivation and optogenetics (47). Moreover, the previous studies of cerebellar TMS in SZ did not explore disturbances in cerebellar timing functions as a possible mechanism by which cerebellar abnormalities may give rise to the symptoms of psychosis. Studies in healthy humans have examined the effects of TMS applied to the cerebellum on timing and time perception (37–39, 60–62). In addition, Singh et al. recently examined the effect of cerebellar transcranial pulsed current stimulation (tPCS), a special type of transcranial direct current stimulation, on time perception in patients with SZ (63). To our knowledge, no studies to date have investigated timing in SZ or other psychiatric disorders using cerebellar TMS.

In this study, we administered iTBS, cTBS, and sham TBS in a double-blind randomized cross-over design in patients with psychosis to explore the role of the cerebellum in psychotic disorders. We measured the effects of the three TBS conditions on time perception (specifically, time interval discrimination) and self-reported clinical symptom severity. We predicted that iTBS, but not sham, would result in acute improvement on a time interval discrimination task and reductions in mood and psychotic symptoms; conversely, we expected that cTBS might result in acute transient worsening in the interval discrimination task and worsening of symptoms.

## 2. Methods

### 2.1. Overview of study design

We conducted a case crossover study in which patients with psychosis (SZ, SZA, or BD) each underwent three sessions of theta burst stimulation (TBS) to the cerebellar vermis in a randomized order: one session of sham TBS, one session of continuous TBS (cTBS), and one session of intermittent TBS (iTBS). See technical details for placebo TMS and blinding below. Participants completed self-ratings of mood and psychosis symptoms and performed the interval discrimination before and after each TMS session. Though the effects of a single session of rTMS are believed to be acute and reversible, with effects usually lasting less than an hour, we separated the sessions by at least 36 h to avoid potential residual carry-over TMS effects from the previous study visit.

### 2.2. Participants

The study was approved by the Mass General Brigham (MGB) institutional review board, which oversees human subjects research at both Massachusetts General Hospital (MGH) and McLean Hospital. All participants provided written informed consent. We recruited male and female patients who had previously participated in research within the McLean Psychotic Disorders Division and had given permission to be contacted about future studies. To be eligible, patients had to be 18–50 years in age, meet criteria for SZ, SZA, or BD using the Structured Clinical Interview for DSM-IV (SCID) during prior participation in research, and be on a stable psychiatric medication regimen for at least 1 month prior to and during study participation. In addition, for neuronavigation, we recruited only patients who already had a structural brain MRI on file from previous participation in research.

Participants were excluded if they had any change in psychiatric medications within a month prior to and during study participation; had been diagnosed with intellectual disability; had been deemed to have legal or mental incompetency; met criteria for a DSM-IV-TR substance abuse or dependence within the prior 3 months; had a significant medical or neurological illness; had a prior neurosurgical procedure; had a history of seizures; were treated with electroconvulsive therapy or clinical TMS within the prior 3 months; previously participated in a cerebellar TMS study; had an implanted cardiac pacemaker; had conductive, ferromagnetic or other magnetic-sensitive metals implanted in the head or neck or that were non-removable and within 30 cm of the treatment coil (e.g., aneurysm clips or coils, carotid or cerebral stents, metallic devices implanted in the head, facial tattoos or permanent makeup using metallic ink, etc.); or were pregnant.

At the first study visit, prior to the first TMS administration, we characterized patients’ baseline clinical characteristics by administering the Positive and Negative Syndrome Scale (PANSS), Young Mania Rating Scale (YMRS), Montgomery-Asberg Depression Rating Scale (MADRS), Psychotic Symptom Rating Scale (PSYRATS), and North American Adult Reading Test (NAART). We also collected demographic (age, sex, race/ethnicity, education level) and medication information. We report antipsychotic medication dosages in chlorpromazine (CPZ) equivalent doses.

### 2.3. Transcranial magnetic stimulation parameters and procedures

All TMS procedures took place at the MGH Laboratory for Neuropsychiatry and Neurostimulation in Boston, MA. Stimulation was delivered using a MagVenture^®^ MagPro X100 stimulator and the Cool DB-80 Active/Placebo figure-of-eight bent coil (MagVenture, Denmark). This coil has a 120° angle designed to stimulate deeper structures. We administered TBS at 100% of active motor threshold (AMT) over the anterior tibialis, a lower extremity muscle which has its primary motor cortical representation deeper in the midline (interhemispheric fissure), more representative of the depth of our cerebellar target than the superficial dorsal representation of the hand. This strategy has been used safely and effectively in previous cerebellar TBS studies [see Hurtado et al. (52) for a detailed discussion]. The AMT was defined as the minimum intensity to elicit a motor-evoked potential greater than 200 μV peak-to-peak, in at least 50% of the trials (3 out of 6) while sustaining a voluntary muscle contraction of approximately 25% of the maximum. We measured the AMT at the first study visit only, but in cases when more than two weeks had passed from the initial AMT measure, we measured it again. Continuous TBS consisted of 3 biphasic pulses delivered at 50 Hz, with these bursts repeated every 200 ms (5 Hz) for 40 s, resulting in a total of 600 pulses per session. Intermittent TBS also applied 600 pulses but over 190 s with cycles of 2 s of stimulation followed by an 8 s pause. Sham sessions used cTBS for half of the patients and iTBS for the other half in a randomized order.

The dual active-placebo Cool DB-80 coil is designed in an X-shape, with 2 bent figure-of-eight coils in opposing configurations. Both sides are visually identical, but the placebo side is magnetically shielded. This design allows for transmitting the vibration of the magnetic pulses only (i.e., auditory and sensory stimulation), without electromagnetic neuromodulation. In addition, a pair of electrodes for skin stimulation was also placed immediately below the hairline under the coil to emulate the tactile sensation generated by the electromagnetic fields over the soft tissue, muscle, and peripheral nerve endings. Electrodes were placed in all sessions but were only active in sham sessions. Using research blinding software embedded in the stimulator, the TMS technician entered a multinumeric code that determined if the session was active or sham, and the stimulator then required technicians to use the corresponding side of the coil while keeping them blinded. Hence, placebo TMS was procedurally identical to the active conditions but used the shielded side of the coil designed to only induce the nonspecific sensory effects of TMS (auditory and somatosensory activation) without the neuromodulatory magnetic fields. At the end of each of the three study visits, we assessed the efficacy of the blind by asking participants to indicate what TMS condition—sham or active—they thought they received that day.

**Figure 1:**
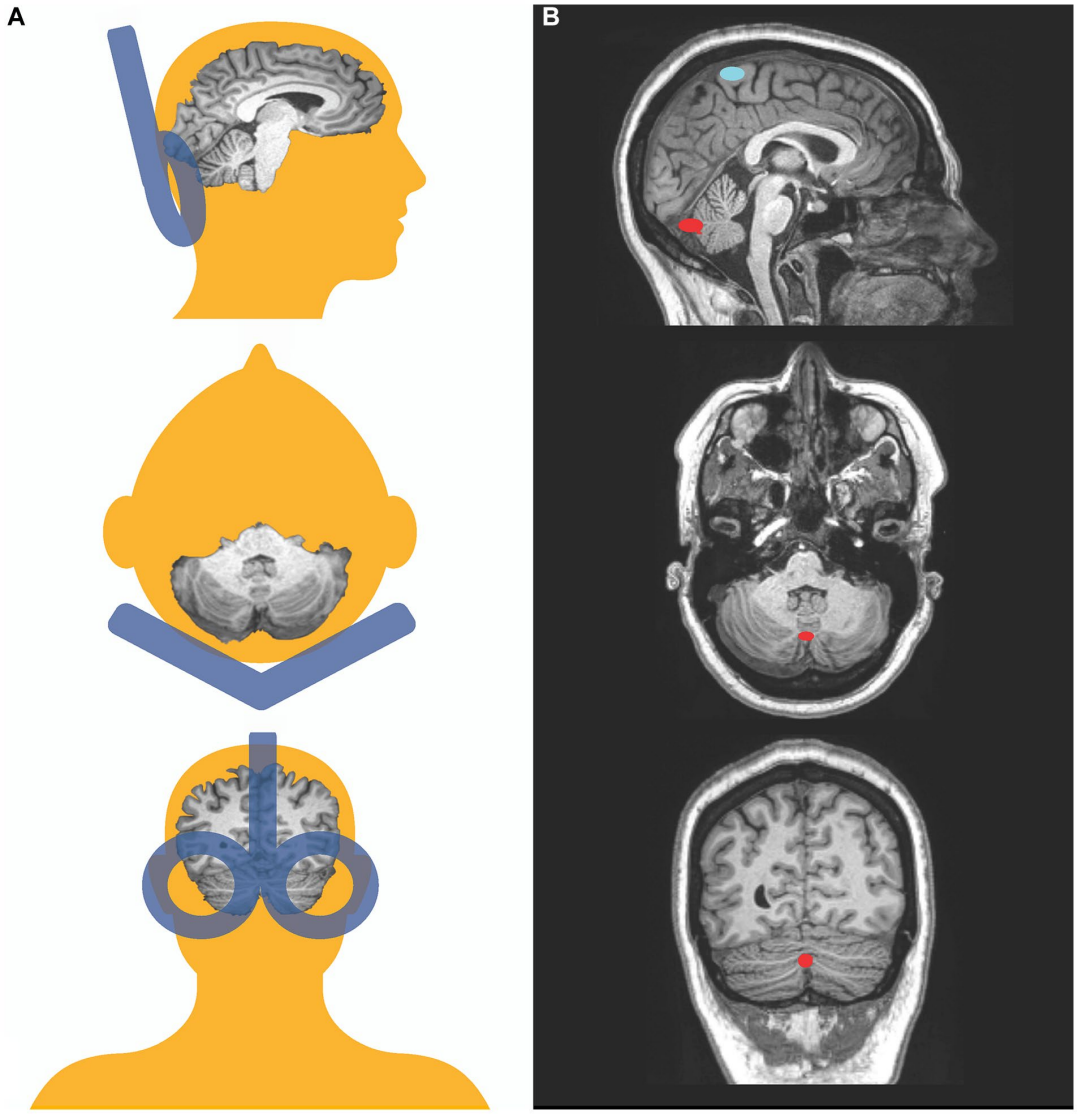
Transcranial magnetic stimulation targets. Schematic representation of coil positioning and target location in this study. Posterior, superior, and lateral views **(A)**. Coronal, transversal, and mid-sagittal MRI slices of a participant depicting the target on the vermis in red and the motor threshold target, the representation of the tibialis anterior, in blue **(B)**.

TBS was administered with the participant sitting upright in a comfortable TMS chair. The TMS coil placed over the occiput with the handle pointing upward. We used stereotactic neuronavigation with infrared optical tracking (Localite, Germany) to identify the cerebellar vermis as the TBS target and to monitor the position of the coil throughout the stimulation session. Using a T1-weighted structural MRI for each participant, we identified the most posterior portion of the cerebellar vermis (midline) and the coil position for the shortest scalp to vermis distance. We targeted the vermis of the cerebellum because postmortem (64–66) and neuroimaging studies (67–74) have reported abnormalities in the cerebellar vermis of patients with SZ. While lateral hemispheric regions of the cerebellum such as Crus I and II—which have functional connections with higher order association areas such as the default, frontoparietal/control, and salience/ventral attention networks (75–77)— are also implicated in psychosis pathophysiology [e.g., (78, 79)], we targeted the vermis in the medial cerebellum because studies have shown that rTMS applied to the medial cerebellum can modulate time perception in healthy individuals (38, 60, 62). Importantly, in people with SZ, there is evidence of hypoactivity in the cerebellar vermis during performance of a timing task, with vermal activation negatively associated with time processing deficits (43). While this same study found timing deficits to also be associated with altered brain activity in Crus I and II, the findings in Crus I and II were in the opposite direction (i.e., Crus I/II hyperactivations), and activity in these lateral cerebellar regions was not correlated with the severity of timing deficits (43). We targeted the posterior vermis (lobules VI-X), which is believed to subserve cognitive and affective functions (vs. the anterior vermis, comprised of lobules I-V, which is associated with somatomotor functions). Also from a practical standpoint, the posterior vermis (especially lobules VII-VIII) is closer to the skull surface, making it better positioned to receive direct TMS stimulation. Using the Localite TMS neuronavigation system, we co-registered the participant’s head to his or her own MRI, and placed the TMS coil over the scalp position that allows direct stimulation of the vermal lobules VII-VIII in the mid-sagittal plane (Figure 1). We assessed the accuracy of the coil placement before and during stimulation with a movement tolerance of 5 mm.

### 2.4. Interval discrimination task

The interval discrimination task (IDT) (80) requires participants to perform time interval comparisons. In each trial, a pair of two tones separated by 1,200 ms (standard interval) is followed by a 1,000 ms delay (interstimulus interval), after which a second comparison pair of tones (experimental interval) is presented. The duration of the experimental interval (time interval separating the second pair of tones) is either *equal* to (1,200 ms, E-condition), 120 ms *longer* than (1,320 ms, L-condition), or 120 ms *shorter* than (1,080 ms, S-condition) the standard interval. The tones for all conditions were 700 Hz in frequency and 50 ms in duration, presented binaurally via headphones. Studies of time interval discrimination have utilized a variety of different structural parameters, and have generated mixed results (81). In selecting the task parameters for the current study, we adopted the methods described by Papageorgiou et al. (80) so as to enable comparison of our findings with previous studies of interval discrimination in SZ. Providing support for the IDT version used in this study, temporal sensitivity has been shown to be higher for auditory than visual intervals (81); unaffected by the presentation of filled (stimulus presented continuously) vs. empty intervals (only the onset and offset are marked, with a silent period in between) as used in the current task (81); and similar across base durations ranging from 200 ms to 1,400 ms (82). Furthermore, research has shown that people with SZ have temporal processing deficits across a range of interstimulus intervals (300 ms to 3,000 ms) (83), which includes the interstimulus interval of 1,000 ms used in our study. Additionally, the inclusion of variable foreperiods (time from completion of the participant’s response on the preceding trial to the onset of the first stimulus presentation on the following trial) can influence both interval discrimination and reaction time by varying the level of preparation that a participant has to respond to the stimulus in the subsequent trial (84). However, the foreperiod length was held constant in our task.

Participants performed the task on a MacBook Pro laptop computer with the task presented using Superlab v5.0 (Cedrus Corporation, San Pedro, CA). Participants were visually cued with a fixation cross at the start of each trial. The words, “Pair 1” were shown on the computer screen while participants were presented with the first pair of tones, and “Pair 2” shown while participants were presented with the second pair of tones. After each trial, participants were instructed to press “e,” “l,” or “s” on the computer keyboard to indicate if the interval between the second pair of tones was equal, longer, or shorter, respectively, than the interval between the first pair. There were an equal number of equal, longer, and shorter trials, and trials were presented in pseudorandom order. The task was designed so that participants responded to all trials; the program did not advance to the subsequent trial without a keyboard response. Participants completed 15 trials during each pre-or post-TMS session for a total of up to 90 trials across the three study visits. Prior to each IDT session, participants performed a practice run consisting of six trials. The primary outcomes for this task were overall accuracy (percent of correct responses) and reaction time (RT). Test–retest reliability for IDT accuracy, as measured by the intra-class correlation (ICC) of accuracy scores across the three pre-TMS sessions, was fair (ICC 0.51, 95% CI 0.30–0.72). The ICC for mean RT was moderately high (ICC 0.77, 95% CI 0.65–0.85). These test–retest results suggest that our IDT data have fair to good reliability across study visits (separated by 36 or more hours).

### 2.5. Self-rated mood and psychotic symptoms

At each of the three study visits, we assessed both clinical and behavioral measures before and immediately following TMS administration. For clinical symptoms, we instructed participants to indicate on a 0-to-100 point visual analog scale (VAS) their current level of depressed mood, anxiety, elevated mood, auditory hallucinations (AH), visual hallucinations (VH), paranoid ideation (PI), ideas/delusions of reference (IOR), and delusions of control (Supplementary Table S1). VAS’s allow participants to easily and rapidly rate the intensity of subjective measures. Participants indicated their ratings for the above mood and psychotic symptoms in a computerized survey custom-designed using Research Electronic Data Capture (REDCap) (85) hosted at MGB. The slider was originally positioned in the middle of the VAS at a score of 50 (“moderately”). Participants were instructed to move the position of the slider to set a response. A rating of 0 indicates the absence of a symptom (e.g., “not at all”), while 100 indicates high symptom severity (e.g., “the most depressed I have ever felt”). We included mood symptoms because they are core features of BD and SZA, and are also observed in SZ. We included the psychotic symptoms that we did because of their relative accessibility by patient self-report (vs. thought disorder or bizarre behavior) and because we considered these to be more amenable to acute modulation by a single TMS session (vs. negative symptoms, which are relatively persistent and trait-like). For these pre-and post-TMS symptom measurements, we opted to use brief patient self-ratings rather than more widely used and more comprehensive clinician-administered standardized assessment tools (e.g., PANSS, YMRS, MADRS, which we used for baseline clinical characterization) because of the limited window of time we had to assess the effects of TMS and the lack of psychometric validity of these clinical tools to capture rapid changes over minutes. The effects of a single session of TBS are acute and reversible, usually receding in less than an hour, and this narrow window of time limited the use of standardized measures, which take time to administer. To aid in the interpretation of VAS findings, we explored each item’s convergent validity (by calculating Spearman correlations between baseline VAS scores from the first study visit and data from validated symptom measures, collected from the same study visit) and test–retest reliability (by calculating the intra-class correlation of each VAS item across the three pre-TMS sessions). The VAS items for depressed mood, anxiety, AH, VH, and PI showed acceptable validity (Supplementary Table S2) and test–retest reliability (Supplementary Table S3). As there was low evidence for convergent validity, test–retest reliability, or both for elated mood, IOR, and delusions of control, we do not report the results for these three VAS items.

### 2.6. Statistical analyses

The reaction time of single trials was introduced into a generalized linear model with mixed effects (GLMM) with a gamma distribution, modeled using the glmer function of the lme4 package in R software (v1.0.136). There were initially 1,800 trials in the completers-only data (20 participants, 90 trials each), and 2,055 trials in the dataset with all 26 participants. Three trials (from 2 participants) with a reaction time of zero, reflecting that there was zero time for stimulus encoding or response execution, were considered invalid and excluded from analysis. We also excluded outlier data, i.e., reaction times greater than 3 standard deviations above the mean, so that very slow reaction times at the right tail of the gamma distribution would not severely distort the means. There were 33 such outliers in the completers-only data (where the outlier threshold was RT > 5171.99 ms) and 39 outliers in the all-participant data (threshold RT > 5503.93 ms), resulting in 1,764 and 2,013 analyzed trials, respectively. In both the completers-only and all-participant datasets, chi-square tests showed that there were no significant differences in the proportion of outliers (excluded trials) before and after TMS, by condition (iTBS vs. cTBS vs. sham), or by session (pre-iTBS, post-iTBS, pre-cTBS, post-cTBS, pre-sham, post-sham) (all *p*-values >0.05).

We have previously shown that the gamma distribution is particularly well suited to modeling reaction times (86, 87). The GLMM distributional assumptions were validated using the fitdist and gofstat functions in R, which compute the goodness-of-fit statistics for parametric distributions. GLMMs are powerful, flexible modeling strategies for estimating the generalizability of experimental findings. The ability to account for correlated observations (longitudinal measures collected for each subject are non-independent) while also explicitly accounting for interindividual variation in primary effects of interest makes these modeling approaches well-suited for our repeated-measures cross-over design. In particular, considering random effects terms accounts for the possibility that, independent of experimental manipulation, each participant may have a different baseline performance or learning rate. This approach ensures that our observed results are not solely attributed to random variations in the tested cohort, particularly given the relatively small sample size. In addition to comparing the least square (LS) means for reaction times, we assessed reaction time variability by analyzing the coefficient of variation (CV), computed by dividing the standard deviation of the reaction times by the mean reaction time. We calculated the CV of each of the six test sessions for each participant and used mixed effects linear regression models with restricted maximum likelihood (REML) estimation to model the CV data.

Task accuracy (percentage of correct responses) was modeled using a generalized logistic regression with mixed effects and a binomial distribution. Subject ID was included as a random effect to account for baseline differences between subjects, while time points (post- and pre-simulation), stimulation type (sham TBS, iTBS, cTBS), and the interactions between them were included as fixed effects. *Post hoc* tests were performed using the “lsmeans” function, which corrects for multiple comparisons using Bonferroni correction and compares the means of the least squares for each fit. Coefficients were considered significant when *p* < 0.05 (two-tailed). Effect sizes were calculated for statistically significant IDT results using Cohen’s *d* for paired samples.

To assess whether IDT accuracy for each participant was better than chance levels, we conducted binomial tests to identify good IDT performers [similar to previously described methods (80)], testing for each participant the null hypothesis that their performance accuracy was no better than 0.33 (accounting for three possible IDT responses, i.e., shorter, longer, and equal time intervals). Binomial tests were conducted for only pre-TBS trials, as the goal was to assess if IDT performance at baseline, excluding potential TBS effects, was better than chance.

To analyze the visual analog scale (VAS) mood and psychotic symptom scores, we calculated the change in pre-and post-TMS VAS scores (Δ _post-pre_). As the VAS scores did not follow a normal distribution, we performed Friedman’s non-parametric repeated measures ANOVA tests for each of the eight VAS measures to test the null hypothesis that at least one of the conditions (iTBS, cTBS, sham) is different. Given the exploratory nature of this analysis, we set the significance threshold at *p* < 0.05, two-sided, without correcting for multiple comparisons. Statistically significant results from Friedman’s tests were followed by post-hoc pairwise testing using the Wilcoxon signed rank test.

Finally, we evaluated the effectiveness of participant blinding to TBS condition. The main concern here is that sham stimulation may not produce the same experience as active TBS. Therefore, at the end of each of the three study visits, participants completed a simple survey to indicate what condition (active vs. sham) they thought they received that day. We conducted chi-square tests to determine if there were any differences by actual TBS condition or by session number (first, second, or third study visit).

## 3. Results

### 3.1. Participant characteristics

**Figure 2:**
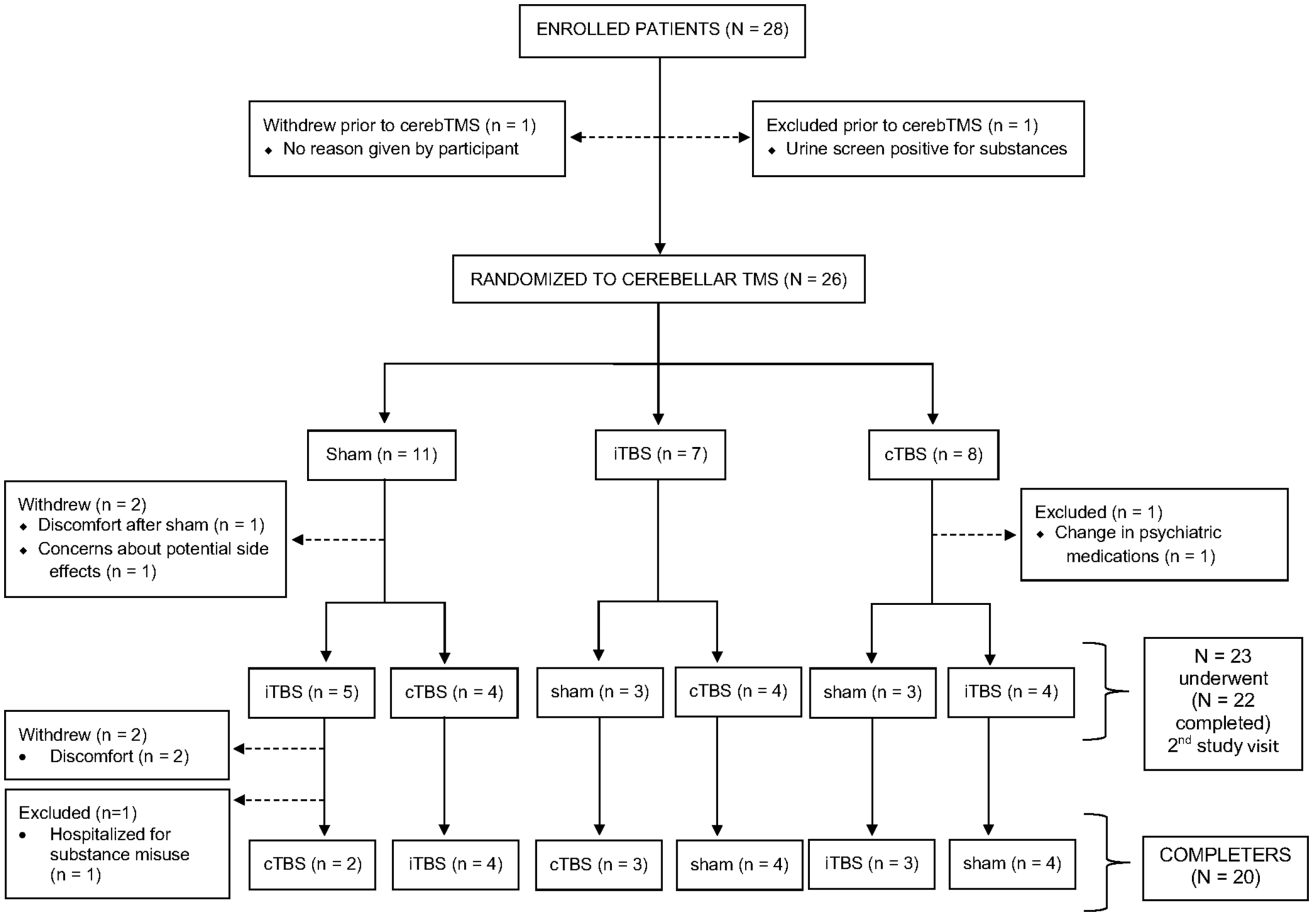
Participant flow diagram. Of 28 participants who enrolled in the study, 26 underwent at least one session of transcranial magnetic stimulation (iTBS, cTBS, or sham) and 20 completed all three sessions.

We enrolled 28 patients with psychotic disorders (6 SZ, 12 SZA, 10 BD). Twenty-six (5 SZ, 12 SZA, 9 BD) underwent at least one session of TMS. Twenty patients completed all three TMS sessions (4 SZ, 9 SZA, 7 BD). Of the eight participants who did not complete the study, three were excluded (1 SZ, 1 SZA, 1 BD) and another five withdrew (1 SZ, 2 SZA, 2 BD) prior to study completion. See flow diagram (Figure 2) for the reasons for exclusions and withdrawals. We report findings from the 20 patients who completed all three TMS sessions (per protocol analysis). We aimed to have 20 completers and focus on completer analysis to avoid biases driven by unbalanced data given the relatively small sample size. Nevertheless, results from all 26 participants who completed at least one study visit (intention-to-treat analysis, though this is a mechanistic and not a therapeutic study) are presented in the Supplementary materials.

See Table 1 for demographic and clinical characteristics of our sample. The twenty completers (4 SZ, 9 SZA, 7 BD) were not significantly different from the eight non-completers (2 SZ, 2 SZA, 3 BD) with respect to age, sex, race/ethnicity, educational level, and estimated IQ. Non-completers seemed to have less severe psychopathology, as evidenced by numerically lower PANSS, YMRS, and MADRS scores; however, the differences between completers and non-completers on these clinical measures were not statistically significant. Similarly, there was no significant difference in the chlorpromazine equivalent antipsychotic doses, and the percentages of patients on antipsychotic and mood stabilizing medications were comparable between the two groups.

**Table 1:** Clinical and demographic characteristics.

	All patients	Completers	Non-completers^a^	Test statistic^b^	*p*-value^b^
Sample size	*N* = 28	*n* = 20	*n* = 8	–	–
Diagnoses, No. (%)				Fisher’s exact	*p* = 1.000
Schizophrenia (SZ)	6 (21.4%)	4 (20.0%)	2 (25.0%)^a^	–	–
Schizoaffective disorder (SZA)	12 (42.9%)	9 (45.0%)	3 (37.5%)	–	–
Psychotic bipolar disorder (BD)	10 (35.7%)	7 (35.0%)	3 (37.5%)^a^	–	–
Age, mean ± SD (range), y	31.8 ± 7.6 (19–48)	31.9 ± 7.8 (19–48)	31.6 ± 7.4 (23–42)	*t* = −0.0698	*p* = 0.945
Female, No. (%)	13 (46.4%)	10 (50%)	3 (37.5%)	Fisher’s exact	*p* = 0.686
Race/Ethnicity				Fisher’s exact	*p* = 0.643
White, Non-Hispanic	21 (75.0%)	15 (75.0%)	6 (75.0%)	–	–
White, Hispanic/Latino	1 (3.6%)	1 (5.0%)	0 (0.0%)	–	–
Black/African-American	2 (7.1%)	2 (10.0%)	0 (0.0%)	–	–
Asian	3 (10.7%)	1 (5.0%)	2 (25.0%)	–	–
Mixed	1 (3.6%)	1 (5.0%)	0 (0.0%)	–	–
Completed education, No. (%)				Fisher’s exact	*p* = 0.154
High school/GED	6 (21.4%)	6 (30.0%)	0 (0.0%)	–	–
Part-college or 2 years college	10 (35.7%)	8 (40.0%)	2 (25.0%)	–	–
College/bachelor’s degree	8 (28.6%)	4 (20.0%)	4 (50.0%)	–	–
Graduate/professional school	4 (14.3%)	2 (10.0%)	2 (25.0%)	–	–
Estimated IQ^c^, mean ± SD					
Verbal IQ	118.3 ± 10.4	116.5 ± 10.8	122.9 ± 8.2	*t* = 1.3995	*p* = 0.175
Performance IQ	114.5 ± 4.9	113.6 ± 5.1	116.6 ± 3.9	*t* = 1.4009	*p* = 0.175
Full scale IQ	118.7 ± 9.1	117.1 ± 9.5	122.7 ± 7.2	*t* = 1.3998	*p* = 0.175
PANSS total score, mean ± SD	41.0 ± 27.3	45.5 ± 23.6	29.9 ± 34.1	*t* = −1.3939	*p* = 0.175
Positive	9.8 ± 7.3	11.1 ± 6.9	6.5 ± 7.7	*t* = −1.5341	*p* = 0.137
Negative	10.4 ± 7.3	11.4 ± 6.5	8.0 ± 9.0	*t* = −1.1145	*p* = 0.275
General psychopathology	20.9 ± 13.5	23.1 ± 11.2	15.4 ± 17.6	*t* = −1.3834	*p* = 0.178
PSYRATS-AH, mean ± SD	4.1 ± 9.4	4.3 ± 9.3	3.6 ± 10.3	*t* = −0.1690	*p* = 0.867
YMRS, mean ± SD	7.3 ± 9.3	7.6 ± 9.8	6.6 ± 8.8	*t* = −0.2326	*p* = 0.818
MADRS, mean ± SD	10.9 ± 11.2	12.8 ± 11.2	6.3 ± 10.4	*t* = −1.4136	*p* = 0.169
CPZ equivalent dose, mean ± SD (range), mg/day	242.4 ± 311.6 (0–1,200)	251.7 ± 317.3 (0–1,200)	219.3 ± 316.9 (0–900)	*t* = −0.2437	*p* = 0.809
Taking antipsychotic drug	17 (60.7%)	12 (60.0%)	5 (62.5%)		
Taking mood stabilizer	14 (50.0%)	10 (50.0%)	4 (50.0%)		
Taking either antipsychotic or mood stabilizer	23 (82.1%)	17 (85.0%)	6 (75.0%)		
Not taking any psychotropic drug	4 (14.3%)	3 (15.0%)	1 (12.5%)		

a Two non-completers either withdrew (1 BD) or were excluded (1 SZ) prior to TBS randomization and do not contribute any results data.

b Test statistics and *p*-values are from a comparison of completers vs. non-completers, using a significance threshold of *p* < 0.05. All *t*-tests are 2-sided.

c Estimated intelligence quotient (IQ) estimated using the North American Adult Reading Test (NAART); NAART data are missing from 3 patients (2 completers, 1 non-completer).

GED, general educational development test; IQ, intelligence quotient; PANSS, positive and negative syndrome scale; PSYRATS-AH, psychotic symptom rating scale, auditory hallucinations subscale; YMRS, Young mania rating scale; MADRS, montgomery-asberg depression rating scale; CPZ, chlorpromazine.

Our protocol involved separating TMS visits by a minimum of 36 h to avoid any potential residual effects of TMS from the previous study visit. Including all participants, the mean number of days between the first and second TMS sessions and between the second and third TMS sessions was 6.6 ± 4.6 (range 3–19) and 5.2 ± 4.9 (range 3–22), respectively. Participants who completed all three study visits did so within a one-month time frame (mean 11.6 ± 6.6, range 4–28 days).

### 3.2. Changes in interval discrimination task performance before and after TMS

**Figure 3:**
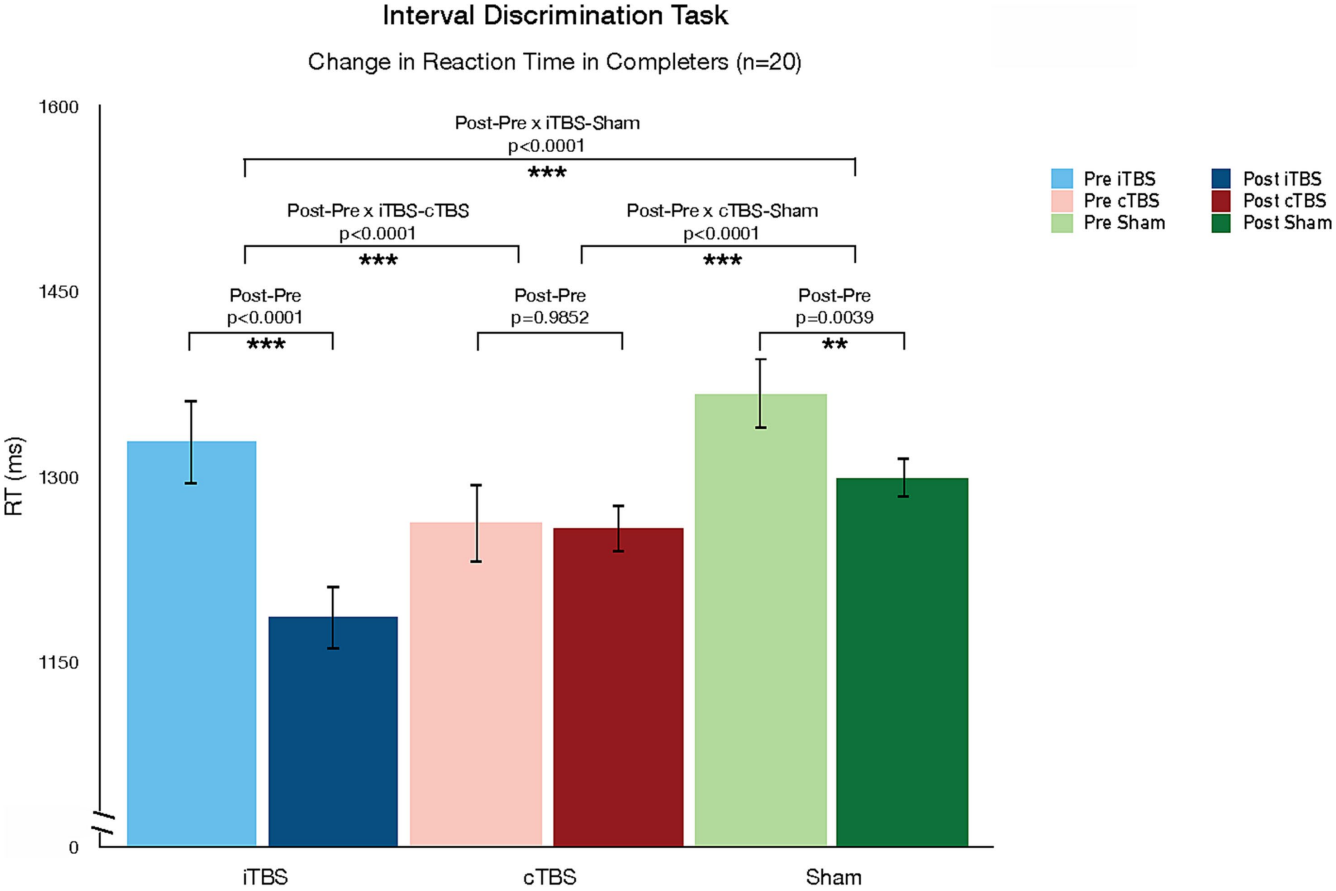
Changes in Interval Discrimination Task (IDT) reaction time pre-and post-TBS. TBS protocols differed in their effect on reaction time in the interval discrimination task. While iTBS reduced reaction time, cTBS increased time.

Table 2 shows the LS-means for each pre-and post-TBS test session. The LS-means are in the range of the reaction times reported in an interval discrimination study conducted in a sample of healthy individuals (88). Analysis of the reaction times showed a significant decrease after iTBS vs. Sham (LS-mean difference = −73.3, *p* < 0.0001), after iTBS vs. cTBS (LS-mean difference = −137.6, *p* < 0.0001), and after Sham vs. cTBS (LS-mean difference = −64.4, *p* < 0.0001) (Figure 3). The corresponding effect sizes, as measured by Cohen’s *d* for paired samples, were *d* = 1.62 (iTBS vs. sham), *d* = 2.03 (iTBS vs. cTBS), and *d* = 1.33 (sham vs. cTBS), indicating large effects. The LS-mean of pre-cTBS reaction times is numerically lower than the LS-means of pre-iTBS and pre-sham reaction times; however, the Kruskal-Wallis test indicated that the reaction times from the three pre-TBS sessions were not statistically significantly different (*p* = 0.794). Analysis of the reaction time coefficient of variation showed no significant effect of TBS condition, pre- vs. post-TBS session, or their interaction on reaction time variability.

**Table 2:** Least square (LS) means for pre-and post-TBS conditions (*n* = 20 Completers).

TBS condition	Time	LS-mean	Standard error
iTBS	Pre	1,328 ms	34.6
	Post	1,186 ms	25.6
cTBS	Pre	1,262 ms	31.1
	Post	1,258 ms	19.1
Sham	Pre	1,367 ms	28.2
	Post	1,299 ms	16.3

We did not observe any significant effects of TBS condition on IDT performance accuracy (Supplementary Tables S4, S5). Binomial tests indicated that 12 of the 20 completers (60%) were good IDT performers, i.e., performing the task better than chance; this group consisted of 2 SZ (50% of SZ), 6 SZA (67% of SZA), and 4 BD (57% of BD) patients. Among all 26 participants, 14 participants including 2 with SZ (40% of 5 SZ with data), 7 with SZA (58% of 12 SZA), and 5 with BD (56% of 9 BD with data) performed the IDT with better-than-chance accuracy. Spaghetti plots, with each participant color-coded by diagnosis, showing pre-and post-TBS within-subject changes in IDT reaction time and accuracy are shown in Supplementary Figures S1, S2, respectively.

### 3.3. Changes in symptom self-ratings before and after TMS

Friedman’s nonparametric repeated measures ANOVA revealed that the TMS conditions differed significantly in their effects on self-ratings of paranoid ideation (Q = 6.745, *p* = 0.034) (Supplementary Table S6). Note that these effects were not corrected for multiple comparisons, given the exploratory nature of this analysis as stated in the methods section. Post-hoc comparisons showed a significant pairwise difference between cTBS and sham (z = 2.227, *p* = 0.026) so that cTBS improved PI (Supplementary Figure S3). This result did not survive correction for multiple comparisons. The three TMS conditions did not significantly differ in changing VAS scores for all other symptom dimensions, even without correction for multiple comparisons, including depressed mood, anxiety, AH, or VH (though see Supplementary Table S10 and Supplementary Figure S8 for the results from the all-participants analysis, showing uncorrected *p* -values < 0.05 for AH as well as PI). Spaghetti plots of within-subject changes in symptom self-ratings pre-and post-TBS, color-coded by diagnosis, are shown in Supplementary Figure S4.

### 3.4. Assessment of participant blinding

Participants could not easily distinguish sham from active TMS. Across the three visits, there was no significant association between the actual condition and the condition participants thought they received (χ^2^ = 3.96, *p* = 0.138 for completers; χ^2^ = 4.65, *p* = 0.098 for all participants). There was also no significant association between visit number (first, second, or third study visit) and what condition participants guessed (χ^2^ = 3.08, *p* = 0.215 for completers; χ^2^ = 2.18, *p* = 0.337 for all participants).

## 4. Discussion

In this study, we used a randomized double-blind cross-over design to explore the effects of a single session of intermittent (iTBS), continuous (cTBS), and sham TBS targeted to the cerebellar vermis using individualized T1 structural MRI-guided stereotactic neuronavigation on time perception (using the interval discrimination task) and mood and psychotic symptoms in a mixed sample of patients with psychotic disorders. We observed that TBS protocols differed in their effect on reaction time in the interval discrimination task: while iTBS reduced RT, cTBS increased RT relative to sham effects. These changes in RT were not at the expense of changes in task accuracy. In fact, we did not observe any changes in task accuracy associated with TBS protocols. The lack of significant findings with respect to task accuracy does not appear to be due to the inability of our participants to perform the task. Sixty percent of participants who completed the study demonstrated better than chance performance in the IDT at baseline. Our sample performed better than the 20% of SZ good IDT performers in a previous study using 1,200 ms range IDT (80), but worse than the 86% of healthy individuals identified as good performers in the same study. In addition, we observed an effect of TBS on one symptom dimension: cTBS improved paranoid ideation compared to sham, but we did not observe any changes from iTBS. We should note that this effect was not corrected for multiple comparisons, and the finding does not survive correction. No other effects on symptom dimensions were observed.

TBS has well-characterized parameter-dependent (i.e., frequency-dependent) dissociable neurophysiological effects on cortical excitability and neuroplasticity: while iTBS leads to post-stimulation LTP-like increases in cortical excitability, cTBS leads to LTD-like decreases in cortical excitability (89, 90). These effects have been primarily demonstrated in the motor cortex, and while most TBS studies have now targeted non-motor areas, it remains a partial assumption that the physiological impact of TBS parameters on cortical motor physiology translates to non-motor cortical targets and circuits. This assumption carries even more uncertainty as we consider the impact of TBS on the cerebellum (52). The histology of the cerebellar cortex and vermis is significantly different from that of the highly structured multilayer cerebral cortex, and so are the patterns of cerebellar neuronal connectivity. These morphological differences (types of cells, local organization of cells, and distal connections of cells) translate into differences in neurophysiological profiles and states (91, 92). As the effects of device-based neuromodulation techniques, including TMS, have been demonstrated to be heavily state-dependent (86, 93), one should not assume that the patterns of response to TBS observed in the cerebral cortex directly translate to the cerebellum: these principles need to be tested empirically. While our study did not have neurophysiological outcome measures, we did observe a dissociation in the direction of the behavioral effect of TBS as a function of the stimulation frequency (or duty cycle), similar to the physiological effects described in the cerebral cortex: relative to sham effects, iTBS (excitatory in the motor cortex) decreased reaction time on a temporal discrimination task, while cTBS (inhibitory in the motor cortex) increased reaction time. Though reaction time can be modulated by factors other than the perceptual and motor-planning computations required to prepare a response (94), processing speed is a key component of reaction time. In this context, our findings suggest that iTBS improved while cTBS decreased processing speed. Our results thus suggest that cerebellar TBS leads to dissociable frequency-dependent neuromodulatory effects, similar to the effects of TBS in the cerebral cortex. Future studies should continue to explore the parameter space in cerebellar neuromodulation (e.g., comparing different stimulation frequencies) while adding neurophysiological outcome measures to understand the biological basis of this behavioral dissociation, and further characterize the differences and similarities between cerebellar vs. cerebral cortical responses to TMS.

The “cognitive dysmetria” (2, 95, 96) and “dysmetria of thought” psychopathological and pathophysiological models (1, 4) propose that psychotic symptoms are manifestations of dysmetria, or incoordination, of mental activity resulting from cerebellar and/or cerebro-cerebellar circuit dysfunction. Providing support for these models, there is accumulating evidence for abnormalities of cerebellar structure (8, 97, 98), function (2, 5–7, 99, 100), and connectivity (78, 79, 98, 101–115) in psychotic disorders, with some studies suggesting that abnormalities within cerebro-cerebellar circuitry may even precede (109) and predict progression (110, 113) to psychosis. Previous studies in healthy individuals suggest that the medial cerebellum is a suitable site to interfere with time perception using 1 Hz rTMS (38, 60) or cTBS (62). In addition, the IDT is a task that is expected to be sensitive to disruptions in cerebellar functioning: studies suggest that interval-based tasks such as the IDT rely on mechanisms involving the cerebellum (62, 116). In healthy individuals, TBS to the medial cerebellum alters interval discrimination but not relative beat-based timing tasks (62), which appear to depend more on the basal ganglia (116).

We used a version of the IDT that has been shown to be abnormal in SZ (80) and report frequency-dependent dissociable effects of iTBS vs. cTBS. Our results suggest an adaptive role of iTBS (improved reaction time) contrasted with a maladaptive role of cTBS (worsened reaction time) relative to the effects of sham. These data lead to the translational hypothesis that iTBS to the cerebellar midline may be therapeutic for psychotic patients. In particular, it could be therapeutic for symptom domains more directly associated with time perception and temporal discrimination. A more nuanced understanding of the association between temporal discrimination deficits and psychotic symptom domains and dimensions would allow a more precise hypothesis about the potential therapeutic benefit of iTBS. It is important to note that our study was designed as a mechanistic, not therapeutic, study: the effects of a single session of TBS are transient and return to a homeostatic baseline approximately 1 h after stimulation. That said, we demonstrate a behavioral target engagement of potential therapeutic significance for the clinical population of study.

It is notable that sham stimulation alone led to a statistically significant non-specific reduction in RT (one could hypothesize this to be driven, at least partially, by practice effects that made subjects faster even if not more accurate). Multiple factors can affect repeated measures performance in behavioral tasks, some associated with the psychometric properties (e.g., learning effects), some with the experimental setting (e.g., duration of the experiment, which can be associated with fatigue), and some with the population of study (e.g., healthy vs. clinical cohorts). Therefore, the observation of longitudinal changes in behavioral task outcomes under sham stimulation conditions is possible and therefore needs to be measured and appropriately controlled for. Interestingly, the uncontrolled change observed before and after cTBS (within condition) was not significant, but when compared with sham (between conditions) and therefore controlling for non-specific confounders, it revealed a *de facto* significant slowing in RT. This highlights the importance of sham-controlled studies in behavioral TMS research: when compared with the expected non-specific increase in RT captured by the sham condition, cTBS revealed its maladaptive reduction in processing efficiency and speed.

The results in the IDT may contrast with those observed with self-reported symptoms: while the task results conclude that iTBS may be adaptive, we did not observe any positive changes in clinical symptoms after iTBS. Moreover, only one clinical dimension (paranoid ideation) was possibly modulated by TBS, and it was cTBS that improved severity compared to sham (there were no effects associated with iTBS). While the positive effect of cTBS on paranoia may seem contradictory, it is important to highlight that the analysis of symptom severity was not corrected for multiple comparisons and that when correction was applied there were no effects of any TBS condition on any of the symptoms. While we decided to show this uncorrected result, given the small sample size and exploratory nature of the symptom analysis, it is conservative to conclude that while a single session of TBS to the cerebellar midline led to dissociable effects on the reaction time of interval discrimination in psychotic patients, it did not translate into significant effects in symptom severity captured with visual analog scales. Visual analog scales are valid and easy-to-use methods to capture rapid changes in symptom severity, but they are noisy and imperfect clinical outcome measures. Behavioral tasks (like the IDT) are better suited to capture the effects of single-session perturbation studies like ours, but they often reflect specific circuit computations more than syndromal or symptom severity. It is also worth noting that our sample consisted mostly of stable outpatients with low symptom severity as evidenced by the baseline PANSS, YMRS, and MADRS scores, and this may have caused a floor effect in the capacity to modulate VAS clinical outcomes. Finally, while a single session of TMS is known to induce transient but measurable biological and behavioral effects, it may not be sufficient to change symptom severity (not even transiently). Hence the lack of clear effects of a single TMS session on symptom severity assessed with VAS in patients with psychosis should not be interpreted as proof of the lack of therapeutic potential of repeated cerebellar TBS sessions, particularly in light of the reported behavioral results.

### 4.1. Limitations

The strengths of this study include the parametric exploration of the role of cerebellar TBS frequency by including two active TBS conditions and sham, the cross-over design, the use of individualized MRI-guided stereotactic neuronavigation for precise targeting of TBS to the medial cerebellum (i.e., vermis), and the choice of a task (IDT) that captures a cognitive dimension associated with cerebellar function and psychopathology in psychosis. However, this study also had several limitations.

First, there are limitations related to our sample, chief of which is that the sample size of 26 participants (only 20 of whom completed all three visits) is small. While our statistical analysis of the interval discrimination task was able to use more robust statistics, the analyses of secondary clinical outcomes were uncorrected for multiple comparisons and remain quite exploratory. Another limitation is that our sample consisted mostly of stable outpatients with low psychosis symptom severity (particularly among the subset with psychotic BD), which may have caused a floor effect in the capacity to modulate VAS clinical outcomes. Similarly, the mean IQ (full scale IQ 117 among the 20 participants who completed the study) and level of completed education (30% of completers finished college or graduate/professional school) of our participants were relatively high for a psychosis sample, and this may limit the generalizability of our findings. Future studies should examine the degree to which cognitive ability predicts or moderates the response to TBS in psychotic disorders. Furthermore, most patients were medicated, and it is unclear how TBS and medications interact; however, we employed a within-subject crossover design, and medications and their dosages were constant for the duration of the study across the three TBS conditions. Additionally, our psychosis sample was diagnostically heterogeneous, including patients with psychotic BD as well as SZ spectrum disorders. It is recognized that these disorders have substantial genetic and clinical overlap. Indeed, a study that investigated timing abnormalities in both SZ spectrum and BD patients found that the bisection point did not differ across groups, suggesting a similar timing deficit in the patient groups (46). Nevertheless, this was a single study, and the ways in which BD and SZ spectrum disorders differ with respect to cerebellar function and temporal discrimination remain to be determined. Though we provide visualizations of individual-level pre- vs. post changes color-coded by diagnosis (Supplementary Figures S1, S2, S4), the limited sample size of this pilot study restricted our ability to conduct subgroup analyses or to directly compare our outcome measures between diagnostic groups, and we are unable to draw any conclusions about the response to TBS according to specific psychosis diagnoses. Investigating similarities and differences in response to cerebellar stimulation across psychotic disorders would be a valuable area of future research. A final point related to our study sample is that we did not collect data from healthy controls. Future studies of cerebellar timing functions in psychotic disorders should include a healthy control group by which to compare the time discrimination findings of people with psychosis, as well as to enable comparisons with the larger literature on cerebellar timing functions in healthy individuals.

Second, there are limitations related to the timing task we employed. While the interval discrimination task is relatively easy to administer and interpret, and has been implemented in many studies of temporal processing including those focused on SZ (80, 83, 117, 118), the measure of accuracy for each trial is binary (correct or incorrect). Further, we collected data for a limited number of stimulus intervals (1,080 ms, 1,200 ms, and 1,320 ms). A task with a more continuous outcome measure, such as the repetitive finger tapping task (119), while more susceptible to potential motor confounds, may have enabled detection of more subtle changes in timing accuracy and variability before and after TBS. The temporal bisection task—in which participants first encode short and long anchor durations and are then presented with stimuli of intermediate durations which they classify as most similar to either the short or the long anchor interval—has also been used to study time perception in SZ (120, 121). While the response to each trial in the temporal bisection task is also binary (short or long), the proportion of “long” responses can be modeled as a function of stimulus duration (122), and changes in perceived time can be identified by shifts of the bisection point (the duration at which short and long classifications are made with equal probability). While these alternative timing tasks may have provided greater sensitivity to detect more subtle time perception changes in response to a single TMS session, it is important to note that we were able to detect the effects of different TBS conditions on the continuous variable of IDT reaction time, even if we identified no effects of TBS condition on the binary measure of interval discrimination accuracy.

Another important limitation related to our task design is that we did not have a reaction time control task to assess the degree to which the observed effects of TBS on IDT reaction times may be due to effects on motor speed and/or behavioral activation rather than selective effects on IDT processing speed. Similarly, we cannot rule out that TBS effects on attention, working memory, and other cognitive processes—which are commonly impaired in psychotic disorders—may account for some of the reaction time results we observed. Though the issue remains debated, the distinct timing hypothesis proposes that there are two distinct mechanisms for temporal processing, with processing of intervals in the sub-second range involving a sensory/automatic timing mechanism not accessible to cognitive control while temporal processing of supra-second intervals is more cognitively mediated (42, 88, 123). Evidence from functional neuroimaging studies suggests that automatic timing is mediated by supplementary motor area (SMA), sensorimotor cortex, cerebellum, premotor area, thalamus, and basal ganglia, while cognitively mediated timing tasks additionally recruit multi-purpose cognitive circuits within the prefrontal and parietal cortices (124). Our task tested time intervals in the 1,200 ms range. Though 1,200 ms is substantially shorter than the higher interval ranges (3–120 s) that some SZ studies (121, 125–127) have used, our interval range is still in the 1 s range and thus may have been susceptible to cognitive confounds. Thus, even though studies indicate that people with SZ have been shown to have timing deficits across a wide range of tasks, independent of whether tasks used sub-second or suprasecond intervals (42), it cannot be excluded that the differential effects of TBS condition on IDT reaction time could be due, in part, to differential impacts of iTBS, cTBS, and sham TBS on cognitive functioning. Finally, we administered a single version of the IDT across the six testing sessions. Given that this was a case cross-over design, repeated administration of the same task could introduce practice effects. Indeed, RT’s before and after sham TBS did show evidence of practice effects during a single study visit. Importantly, however, we captured these learning effects by including a sham condition, and still showed that the iTBS and cTBS conditions significantly differed from these sham effects.

Third, there are limitations with respect to our symptom measures. Visual analog scales are easy to use measures that allow for the assessment of rapid changes in severity. The five visual analog scales for which we report results (depressed mood, anxiety, AH, VH, and PI) have satisfactory test–retest reliability and convergent validity with standardized measures, but are very noisy. As rapidly effective treatments (including device-based treatments) are being developed, there is a growing need to develop more robust psychometrically validated measures of rapid changes in neuropsychiatric symptom severity. Another limitation is that we did not evaluate negative symptoms, the domain reported to improve with cerebellar TMS in previous studies of SZ (53–57). However, the goal of our study (which was mechanistic and not therapeutic in nature) was to capture immediate changes within the hour following a single session of TBS, and acute changes in negative symptoms which tend to be relatively persistent, trait-like phenomena are more challenging to measure.

Fourth, we did not include any biological markers by which to measure TBS effects. We selected the cerebellum as the target for neuromodulation because of its emerging role as a brain area of scientific interest in psychotic disorders, its relevance to temporal processing, and its accessibility close to the skull surface. However, the cerebellum is only one of several brain areas involved in temporal processing and is unlikely to be the only target for modulating timing deficits in SZ. For example, Walther and colleagues found that in patients with SZ, a single session of cTBS to the right inferior parietal lobule (IPL) improved both gesture performance accuracy and manual dexterity (128), both of which are more complex motor behaviors but ones that involve mechanisms of timing. Importantly, the cerebellum communicates with many distributed brain areas, including prefrontal and parietal cortices, through polysynaptic cerebro-cerebello-thalamo-cerebral (CCTC) circuits (23). It has already been demonstrated that iTBS targeting the medial cerebellum can impact its connectivity with the dorsolateral prefrontal cortex (DLPFC) (56). Similarly, a single session of transcranial pulsed current stimulation to the medial cerebellum during a timing task improved frontal theta oscillations in patients with SZ (63). Conversely, rTMS targeted to the left DLPFC in SZ has been shown to modulate functional connectivity with the cerebellum, thalamus, and other regions within CTCC circuits (129). While it is clear that applying rTMS to one brain area has effects in brain areas that are functionally connected, it remains unclear what neural changes are driving the differential response to TBS effects in the present study. Assessing how cerebellar TBS affects cerebellar physiology and distal connectivity associated with timing and/or psychotic symptoms will be critical in future studies.

Finally, while we used individualized MRI-guided stereotactic target selection, a TMS coil with a 120° angle designed to stimulate deeper structures, and a stimulation intensity with established safety and proven capacity to modulate physiology, behavior, and clinical symptoms, anatomical targeting and dosing remain unresolved problems in cerebellar TMS. The strength of the TMS magnetic field decays rapidly as it moves away from the coil, making TMS a relatively shallow neuromodulatory intervention (48). The cerebellum is a relatively deep structure, with a greater distance to the skull surface than typical cerebral cortical targets. Moreover, there are generally different types of tissues (including a large pool of cerebrospinal fluid) in between the coil and the target, and these anatomical characteristics can be variable across individuals. As we lack electric field modeling studies to understand how all these factors shape the actual topography and intensity of the TMS-induced electric fields across individuals, one should be cautious when making very specific anatomical inferences. Modeling and dose–response studies are urgently needed to accelerate the therapeutic potential of cerebellar TMS.

## 5. Conclusion

In conclusion, we demonstrate a frequency-dependent dissociation between the acute effects of a single session of iTBS vs. cTBS to the cerebellar midline (600 pulses per session, 100% MT of the AMT, a deep 120° bent figure-of-eight coil, and individualized MRI-guided stereotactic neuronavigation) on the speed of response during a time interval discrimination task in patients with psychosis. Specifically, iTBS showed improved reaction time (adaptive) while cTBS led to worsening speed of response (maladaptive). We did not observe any effects of TBS on affective or positive symptoms of psychosis when appropriately controlling for multiple comparisons. The results of this mechanistic behavioral neuromodulation study demonstrate behavioral target engagement in a cognitive dimension of relevance to the psychopathology and pathophysiology of patients with psychosis, and generate testable hypotheses about the potential adaptive therapeutic role of iTBS to the cerebellar midline in this clinical population.

## Data availability statement

The original contributions presented in the study are included in the article/Supplementary materials, further inquiries can be directed to the corresponding author.

## Ethics statement

This study, involving humans, was approved by the Mass General Brigham (MGB) IRB (formerly Partners IRB). The study was conducted in accordance with the local legislation and institutional requirements. The participants provided their written informed consent to participate in this study.

## Author contributions

AS: conceptualization, methodology, formal analysis, writing – original draft, writing – review and editing, and supervision. AH-P: software, investigation, formal analysis, visualization, and writing – review and editing. YR: software, investigation, and project administration. VH and MH: investigation and project administration. BC and DÖ: conceptualization, methodology, resources, and writing – review and editing. JC: conceptualization, methodology, resources, writing – original draft, writing – review and editing, and supervision. All authors contributed to the article and approved the submitted version.

## References

[1] Schmahmann JD. An emerging concept. The cerebellar contribution to higher function. Arch Neurol. (1991) 48:1178–87. doi: 10.1001/archneur.1991.00530230086029 1953406

[2] Andreasen NC O'Leary DS Cizadlo T Arndt S Rezai K Ponto LL. Schizophrenia and cognitive dysmetria: a positron-emission tomography study of dysfunctional prefrontal-thalamic-cerebellar circuitry. Proc Natl Acad Sci U S A. (1996) 93:9985–90. doi: 10.1073/pnas.93.18.9985, PMID: 8790444 PMC38542

[3] Andreasen NC Paradiso S O'Leary DS. "Cognitive dysmetria" as an integrative theory of schizophrenia: a dysfunction in cortical-subcortical-cerebellar circuitry? Schizophr Bull. (1998) 24:203–18. doi: 10.1093/oxfordjournals.schbul.a033321, PMID: 9613621

[4] Schmahmann JD. Dysmetria of thought: clinical consequences of cerebellar dysfunction on cognition and affect. Trends Cogn Sci. (1998) 2:362–71. doi: 10.1016/S1364-6613(98)01218-2, PMID: 21227233

[5] Martin P Albers M. Cerebellum and schizophrenia: a selective review. Schizophr Bull. (1995) 21:241–50. doi: 10.1093/schbul/21.2.241 7631171

[6] Hoppenbrouwers SS Schutter DJ Fitzgerald PB Chen R Daskalakis ZJ. The role of the cerebellum in the pathophysiology and treatment of neuropsychiatric disorders: a review. Brain Res Rev. (2008) 59:185–200. doi: 10.1016/j.brainresrev.2008.07.005, PMID: 18687358

[7] Bernard JA Mittal VA. Dysfunctional activation of the cerebellum in schizophrenia: a functional neuroimaging meta-analysis. Clin Psychol Sci. (2015) 3:545–66. doi: 10.1177/2167702614542463, PMID: 26392921 PMC4574495

[8] Moberget T Doan NT Alnaes D Kaufmann T Cordova-Palomera A Lagerberg TV. Cerebellar volume and cerebellocerebral structural covariance in schizophrenia: a multisite mega-analysis of 983 patients and 1349 healthy controls. Mol Psychiatry. (2018) 23:1512–20. doi: 10.1038/mp.2017.106, PMID: 28507318

[9] Moberget T Ivry RB. Prediction, psychosis, and the cerebellum. Biol Psychiatry Cogn Neurosci Neuroimaging. (2019) 4:820–31. doi: 10.1016/j.bpsc.2019.06.001, PMID: 31495402 PMC8289739

[10] Middleton FA Strick PL. Anatomical evidence for cerebellar and basal ganglia involvement in higher cognitive function. Science. (1994) 266:458–61. doi: 10.1126/science.7939688, PMID: 7939688

[11] Middleton FA Strick PL. Cerebellar projections to the prefrontal cortex of the primate. J Neurosci. (2001) 21:700–12. doi: 10.1523/JNEUROSCI.21-02-00700.2001, PMID: 11160449 PMC6763818

[12] Kelly RM Strick PL. Cerebellar loops with motor cortex and prefrontal cortex of a nonhuman primate. J Neurosci. (2003) 23:8432–44. doi: 10.1523/JNEUROSCI.23-23-08432.2003, PMID: 12968006 PMC6740694

[13] Cho SS Yoon EJ Bang SA Park HS Kim YK Strafella AP. Metabolic changes of cerebrum by repetitive transcranial magnetic stimulation over lateral cerebellum: a study with FDG PET. Cerebellum. (2012) 11:739–48. doi: 10.1007/s12311-011-0333-7, PMID: 22161500

[14] Brodal P. Principles of organization of the corticopontocerebellar projection to crus II in the cat with particular reference to the parietal cortical areas. Neuroscience. (1983) 10:621–38. doi: 10.1016/0306-4522(83)90207-5, PMID: 6316199

[15] Clower DM West RA Lynch JC Strick PL. The inferior parietal lobule is the target of output from the superior colliculus, hippocampus, and cerebellum. J Neurosci. (2001) 21:6283–91. doi: 10.1523/JNEUROSCI.21-16-06283.2001, PMID: 11487651 PMC6763148

[16] Clower DM Dum RP Strick PL. Basal ganglia and cerebellar inputs to “AIP”. Cereb Cortex. (2005) 15:913–20. doi: 10.1093/cercor/bhh190, PMID: 15459083

[17] Sultan F Augath M Hamodeh S Murayama Y Oeltermann A Rauch A. Unravelling cerebellar pathways with high temporal precision targeting motor and extensive sensory and parietal networks. Nat Commun. (2012) 3:924. doi: 10.1038/ncomms1912, PMID: 22735452

[18] Anand BK Malhotra CL Singh B Dua S. Cerebellar projections to limbic system. J Neurophysiol. (1959) 22:451–7. doi: 10.1152/jn.1959.22.4.451 13673296

[19] Snider RS Maiti A. Cerebellar contributions to the Papez circuit. J Neurosci Res. (1976) 2:133–46. doi: 10.1002/jnr.490020204, PMID: 950678

[20] Leiner HC Leiner AL Dow RS. The human cerebro-cerebellar system: its computing, cognitive, and language skills. Behav Brain Res. (1991) 44:113–28. doi: 10.1016/S0166-4328(05)80016-6, PMID: 1751002

[21] Ito M. Movement and thought: identical control mechanisms by the cerebellum. Trends Neurosci. (1993) 16:448–50. doi: 10.1016/0166-2236(93)90073-U, PMID: 7507615

[22] Ramnani N. The primate cortico-cerebellar system: anatomy and function. Nat Rev Neurosci. (2006) 7:511–22. doi: 10.1038/nrn1953, PMID: 16791141

[23] Strick PL Dum RP Fiez JA. Cerebellum and nonmotor function. Annu Rev Neurosci. (2009) 32:413–34. doi: 10.1146/annurev.neuro.31.060407.125606 19555291

[24] Guell X Gabrieli JDE Schmahmann JD. Embodied cognition and the cerebellum: perspectives from the Dysmetria of thought and the universal cerebellar transform theories. Cortex. (2018) 100:140–8. doi: 10.1016/j.cortex.2017.07.005, PMID: 28779872

[25] Marek S Siegel JS Gordon EM Raut RV Gratton C Newbold DJ. Spatial and temporal organization of the individual human cerebellum. Neuron. (2018) 100:977–993.e7. doi: 10.1016/j.neuron.2018.10.010, PMID: 30473014 PMC6351081

[26] Keele SW Ivry R. Does the cerebellum provide a common computation for diverse tasks? A timing hypothesis. Ann N Y Acad Sci. (1990) 608:179–211. doi: 10.1111/j.1749-6632.1990.tb48897.x 2075953

[27] O'Reilly JX Mesulam MM Nobre AC. The cerebellum predicts the timing of perceptual events. J Neurosci. (2008) 28:2252–60. doi: 10.1523/JNEUROSCI.2742-07.2008, PMID: 18305258 PMC6671847

[28] D'Angelo E Casali S. Seeking a unified framework for cerebellar function and dysfunction: from circuit operations to cognition. Front Neural Circuits. (2012) 6:116. doi: 10.3389/fncir.2012.00116 23335884 PMC3541516

[29] Schwartze M Kotz SA. A dual-pathway neural architecture for specific temporal prediction. Neurosci Biobehav Rev. (2013) 37:2587–96. doi: 10.1016/j.neubiorev.2013.08.005, PMID: 23994272

[30] Bares M Apps R Avanzino L Breska A D'Angelo E Filip P. Consensus paper: decoding the contributions of the cerebellum as a time machine. From neurons to clinical applications. Cerebellum. (2019) 18:266–86. doi: 10.1007/s12311-018-0979-5, PMID: 30259343

[31] Koch G Oliveri M Caltagirone C. Neural networks engaged in milliseconds and seconds time processing: evidence from transcranial magnetic stimulation and patients with cortical or subcortical dysfunction. Philos Trans R Soc Lond Ser B Biol Sci. (2009) 364:1907–18. doi: 10.1098/rstb.2009.0018, PMID: 19487193 PMC2685818

[32] Forti L Cesana E Mapelli J D'Angelo E. Ionic mechanisms of autorhythmic firing in rat cerebellar Golgi cells. J Physiol. (2006) 574:711–29. doi: 10.1113/jphysiol.2006.110858, PMID: 16690702 PMC1817727

[33] Solinas S Forti L Cesana E Mapelli J De Schutter E D'Angelo E. Computational reconstruction of pacemaking and intrinsic electroresponsiveness in cerebellar Golgi cells. Front Cell Neurosci. (2007) 1:2. doi: 10.3389/neuro.03.002.2007 18946520 PMC2525930

[34] Johansson F Jirenhed DA Rasmussen A Zucca R Hesslow G. Memory trace and timing mechanism localized to cerebellar Purkinje cells. Proc Natl Acad Sci U S A. (2014) 111:14930–4. doi: 10.1073/pnas.1415371111, PMID: 25267641 PMC4205653

[35] Jirenhed DA Rasmussen A Johansson F Hesslow G. Learned response sequences in cerebellar Purkinje cells. Proc Natl Acad Sci U S A. (2017) 114:6127–32. doi: 10.1073/pnas.1621132114, PMID: 28533379 PMC5468669

[36] Johansson F. Intrinsic memory of temporal intervals in cerebellar Purkinje cells. Neurobiol Learn Mem. (2019) 166:107103. doi: 10.1016/j.nlm.2019.107103, PMID: 31648018

[37] Koch G Oliveri M Torriero S Salerno S Lo Gerfo E Caltagirone C. Repetitive TMS of cerebellum interferes with millisecond time processing. Exp Brain Res. (2007) 179:291–9. doi: 10.1007/s00221-006-0791-1, PMID: 17146647

[38] Lee KH Egleston PN Brown WH Gregory AN Barker AT Woodruff PW. The role of the cerebellum in subsecond time perception: evidence from repetitive transcranial magnetic stimulation. J Cogn Neurosci. (2007) 19:147–57. doi: 10.1162/jocn.2007.19.1.147, PMID: 17214571

[39] Del Olmo MF Cheeran B Koch G Rothwell JC. Role of the cerebellum in externally paced rhythmic finger movements. J Neurophysiol. (2007) 98:145–52. doi: 10.1152/jn.01088.2006, PMID: 17460103

[40] Minichino A Bersani FS Trabucchi G Albano G Primavera M Delle Chiaie R. The role of cerebellum in unipolar and bipolar depression: a review of the main neurobiological findings. Riv Psichiatr. (2014) 49:124–31. doi: 10.1708/1551.16907, PMID: 25000888

[41] Thoenes S Oberfeld D. Meta-analysis of time perception and temporal processing in schizophrenia: differential effects on precision and accuracy. Clin Psychol Rev. (2017) 54:44–64. doi: 10.1016/j.cpr.2017.03.007, PMID: 28391027

[42] Ciullo V Spalletta G Caltagirone C Jorge RE Piras F. Explicit time deficit in schizophrenia: systematic review and meta-analysis indicate it is primary and not domain specific. Schizophr Bull. (2016) 42:505–18. doi: 10.1093/schbul/sbv104, PMID: 26253596 PMC4753592

[43] Losak J Huttlova J Lipova P Marecek R Bares M Filip P. Predictive motor timing and the cerebellar vermis in schizophrenia: an fMRI study. Schizophr Bull. (2016) 42:1517–27. doi: 10.1093/schbul/sbw065, PMID: 27190280 PMC5049535

[44] Bolbecker AR Hong SL Kent JS Forsyth JK Klaunig MJ Lazar EK. Paced finger-tapping abnormalities in bipolar disorder indicate timing dysfunction. Bipolar Disord. (2011) 13:99–110. doi: 10.1111/j.1399-5618.2011.00895.x, PMID: 21320257 PMC3079233

[45] Ciullo V Piras F Banaj N Vecchio D Piras F Sani G. Internal clock variability, mood swings and working memory in bipolar disorder. J Affect Disord. (2022) 315:48–56. doi: 10.1016/j.jad.2022.07.063, PMID: 35907479

[46] Bolbecker AR Westfall DR Howell JM Lackner RJ Carroll CA O'Donnell BF. Increased timing variability in schizophrenia and bipolar disorder. PLoS One. (2014) 9:e97964. doi: 10.1371/journal.pone.0097964, PMID: 24848559 PMC4029800

[47] Parker KL Kim YC Kelley RM Nessler AJ Chen KH Muller-Ewald VA. Delta-frequency stimulation of cerebellar projections can compensate for schizophrenia-related medial frontal dysfunction. Mol Psychiatry. (2017) 22:647–55. doi: 10.1038/mp.2017.50, PMID: 28348382 PMC5873945

[48] Camprodon JA. Transcranial Magnetic Stimulation In: Camprodon JA Rauch SL Greenberg BD Dougherty DD, editors. Psychiatric neurotherapeutics: contemporary surgical & device-based treatments in psychiatry. New York, NY: Humana Press (2016). 165–86.

[49] Sack AT Kohler A Linden DE Goebel R Muckli L. The temporal characteristics of motion processing in hMT/V5+: combining fMRI and neuronavigated TMS. NeuroImage. (2006) 29:1326–35. doi: 10.1016/j.neuroimage.2005.08.027, PMID: 16185899

[50] Huang YZ Edwards MJ Rounis E Bhatia KP Rothwell JC. Theta burst stimulation of the human motor cortex. Neuron. (2005) 45:201–6. doi: 10.1016/j.neuron.2004.12.033 15664172

[51] Suppa A Huang YZ Funke K Ridding MC Cheeran B Di Lazzaro V. Ten years of Theta burst stimulation in humans: established knowledge, unknowns and prospects. Brain Stimul. (2016) 9:323–35. doi: 10.1016/j.brs.2016.01.006, PMID: 26947241

[52] Hurtado-Puerto AM Nestor K Eldaief M Camprodon JA. Safety considerations for cerebellar theta burst stimulation. Clin Ther. (2020) 42:1169–1190.e1. doi: 10.1016/j.clinthera.2020.06.001, PMID: 32674957

[53] Demirtas-Tatlidede A Freitas C Cromer JR Safar L Ongur D Stone WS. Safety and proof of principle study of cerebellar vermal theta burst stimulation in refractory schizophrenia. Schizophr Res. (2010) 124:91–100. doi: 10.1016/j.schres.2010.08.015, PMID: 20817483 PMC3268232

[54] Tikka SK Garg S Sinha VK Nizamie SH Goyal N. Resting state dense Array gamma oscillatory activity as a response marker for cerebellar-repetitive transcranial magnetic stimulation (rTMS) in schizophrenia. J ECT. (2015) 31:258–62. doi: 10.1097/YCT.0000000000000242, PMID: 25923998

[55] Garg S Sinha VK Tikka SK Mishra P Goyal N. The efficacy of cerebellar vermal deep high frequency (theta range) repetitive transcranial magnetic stimulation (rTMS) in schizophrenia: a randomized rater blind-sham controlled study. Psychiatry Res. (2016) 243:413–20. doi: 10.1016/j.psychres.2016.07.023, PMID: 27450744

[56] Brady RO Jr Gonsalvez I Lee I Ongur D Seidman LJ Schmahmann JD. Cerebellar-prefrontal network connectivity and negative symptoms in schizophrenia. Am J Psychiatry. (2019) 176:512–20. doi: 10.1176/appi.ajp.2018.18040429, PMID: 30696271 PMC6760327

[57] Zhu L Zhang W Zhu Y Mu X Zhang Q Wang Y. Cerebellar theta burst stimulation for the treatment of negative symptoms of schizophrenia: a multicenter, double-blind, randomized controlled trial. Psychiatry Res. (2021) 305:114204. doi: 10.1016/j.psychres.2021.114204, PMID: 34587567

[58] Chauhan P Garg S Tikka SK Khattri S. Efficacy of intensive cerebellar intermittent theta burst stimulation (iCiTBS) in treatment-resistant schizophrenia: a randomized placebo-controlled study. Cerebellum. (2021) 20:116–23. doi: 10.1007/s12311-020-01193-9, PMID: 32964381 PMC7508243

[59] Basavaraju R Ithal D Thanki MV Ramalingaiah AH Thirthalli J Reddy RP. Intermittent theta burst stimulation of cerebellar vermis enhances fronto-cerebellar resting state functional connectivity in schizophrenia with predominant negative symptoms: a randomized controlled trial. Schizophr Res. (2021) 238:108–20. doi: 10.1016/j.schres.2021.10.005, PMID: 34653740 PMC8662658

[60] Theoret H Haque J Pascual-Leone A. Increased variability of paced finger tapping accuracy following repetitive magnetic stimulation of the cerebellum in humans. Neurosci Lett. (2001) 306:29–32. doi: 10.1016/S0304-3940(01)01860-2, PMID: 11403950

[61] Fierro B Palermo A Puma A Francolini M Panetta ML Daniele O. Role of the cerebellum in time perception: a TMS study in normal subjects. J Neurol Sci. (2007) 263:107–12. doi: 10.1016/j.jns.2007.06.033, PMID: 17655867

[62] Grube M Lee KH Griffiths TD Barker AT Woodruff PW. Transcranial magnetic theta-burst stimulation of the human cerebellum distinguishes absolute, duration-based from relative, beat-based perception of subsecond time intervals. Front Psychol. (2010) 1:171. doi: 10.3389/fpsyg.2010.00171 21833234 PMC3153783

[63] Singh A Trapp NT De Corte B Cao S Kingyon J Boes AD. Cerebellar Theta frequency transcranial pulsed stimulation increases frontal Theta oscillations in patients with schizophrenia. Cerebellum. (2019) 18:489–99. doi: 10.1007/s12311-019-01013-9, PMID: 30825131 PMC6818969

[64] Weinberger DR Kleinman JE Luchins DJ Bigelow LB Wyatt RJ. Cerebellar pathology in schizophrenia: a controlled postmortem study. Am J Psychiatry. (1980) 137:359–61. doi: 10.1176/ajp.137.3.359, PMID: 7356066

[65] Tran KD Smutzer GS Doty RL Arnold SE. Reduced Purkinje cell size in the cerebellar vermis of elderly patients with schizophrenia. Am J Psychiatry. (1998) 155:1288–90. doi: 10.1176/ajp.155.9.1288, PMID: 9734558

[66] Reyes MG Gordon A. Cerebellar vermis in schizophrenia. Lancet. (1981) 2:700–1. PMID: 6116081 10.1016/s0140-6736(81)91039-4

[67] Rossi A Stratta P Mancini F de Cataldo S Casacchia M. Cerebellar vermal size in schizophrenia: a male effect. Biol Psychiatry. (1993) 33:354–7. doi: 10.1016/0006-3223(93)90324-7, PMID: 8471693

[68] Jacobsen LK Giedd JN Berquin PC Krain AL Hamburger SD Kumra S. Quantitative morphology of the cerebellum and fourth ventricle in childhood-onset schizophrenia. Am J Psychiatry. (1997) 154:1663–9. doi: 10.1176/ajp.154.12.1663, PMID: 9396943

[69] Nopoulos PC Ceilley JW Gailis EA Andreasen NC. An MRI study of cerebellar vermis morphology in patients with schizophrenia: evidence in support of the cognitive dysmetria concept. Biol Psychiatry. (1999) 46:703–11. doi: 10.1016/S0006-3223(99)00093-1, PMID: 10472423

[70] Loeber RT Cintron CM Yurgelun-Todd DA. Morphometry of individual cerebellar lobules in schizophrenia. Am J Psychiatry. (2001) 158:952–4. doi: 10.1176/appi.ajp.158.6.952, PMID: 11384906

[71] Ichimiya T Okubo Y Suhara T Sudo Y. Reduced volume of the cerebellar vermis in neuroleptic-naive schizophrenia. Biol Psychiatry. (2001) 49:20–7. doi: 10.1016/S0006-3223(00)01081-7, PMID: 11163776

[72] Okugawa G Sedvall G Nordstrom M Andreasen N Pierson R Magnotta V. Selective reduction of the posterior superior vermis in men with chronic schizophrenia. Schizophr Res. (2002) 55:61–7. doi: 10.1016/S0920-9964(01)00248-1, PMID: 11955964

[73] Okugawa G Sedvall GC Agartz I. Smaller cerebellar vermis but not hemisphere volumes in patients with chronic schizophrenia. Am J Psychiatry. (2003) 160:1614–7. doi: 10.1176/appi.ajp.160.9.1614, PMID: 12944335

[74] Joyal CC Pennanen C Tiihonen E Laakso MP Tiihonen J Aronen HJ. MRI volumetry of the vermis and the cerebellar hemispheres in men with schizophrenia. Psychiatry Res. (2004) 131:115–24. doi: 10.1016/j.pscychresns.2003.09.003, PMID: 15313518

[75] Xue A Kong R Yang Q Eldaief MC Angeli PA DiNicola LM. The detailed organization of the human cerebellum estimated by intrinsic functional connectivity within the individual. J Neurophysiol. (2021) 125:358–84. doi: 10.1152/jn.00561.2020, PMID: 33427596 PMC7948146

[76] Buckner RL Krienen FM Castellanos A Diaz JC Yeo BT. The organization of the human cerebellum estimated by intrinsic functional connectivity. J Neurophysiol. (2011) 106:2322–45. doi: 10.1152/jn.00339.2011, PMID: 21795627 PMC3214121

[77] Halko MA Farzan F Eldaief MC Schmahmann JD Pascual-Leone A. Intermittent theta-burst stimulation of the lateral cerebellum increases functional connectivity of the default network. J Neurosci. (2014) 34:12049–56. doi: 10.1523/JNEUROSCI.1776-14.2014, PMID: 25186750 PMC4152606

[78] Shinn AK Baker JT Lewandowski KE Ongur D Cohen BM. Aberrant cerebellar connectivity in motor and association networks in schizophrenia. Front Hum Neurosci. (2015) 9:134. doi: 10.3389/fnhum.2015.00134 25852520 PMC4364170

[79] Shinn AK Roh YS Ravichandran CT Baker JT Ongur D Cohen BM. Aberrant cerebellar connectivity in bipolar disorder with psychosis. Biol Psychiatry Cogn Neurosci Neuroimaging. (2017) 2:438–48. doi: 10.1016/j.bpsc.2016.07.002, PMID: 28730183 PMC5512437

[80] Papageorgiou C Karanasiou IS Kapsali F Stachtea X Kyprianou M Tsianaka EI. Temporal processing dysfunction in schizophrenia as measured by time interval discrimination and tempo reproduction tasks. Prog Neuro-Psychopharmacol Biol Psychiatry. (2013) 40:173–9. doi: 10.1016/j.pnpbp.2012.07.017, PMID: 23367507

[81] Rammsayer TH. The effects of type of interval, sensory modality, base duration, and psychophysical task on the discrimination of brief time intervals. Atten Percept Psychophys. (2014) 76:1185–96. doi: 10.3758/s13414-014-0655-x, PMID: 24596081

[82] Rammsayer T Ulrich R. The greater temporal acuity in the reminder task than in the 2AFC task is independent of standard duration and sensory modality. Can J Exp Psychol. (2012) 66:26–31. doi: 10.1037/a0025349, PMID: 21910520

[83] Davalos DB Kisley MA Ross RG. Effects of interval duration on temporal processing in schizophrenia. Brain Cogn. (2003) 52:295–301. doi: 10.1016/S0278-2626(03)00157-X, PMID: 12907174

[84] Laflamme V Zakay D Gamache PL Grondin S. Foreperiod and range effects on time interval categorization. Atten Percept Psychophys. (2015) 77:1507–14. doi: 10.3758/s13414-015-0937-y, PMID: 26022698

[85] Harris PA Taylor R Thielke R Payne J Gonzalez N Conde JG. Research electronic data capture (REDCap)—a metadata-driven methodology and workflow process for providing translational research informatics support. J Biomed Inform. (2009) 42:377–81. doi: 10.1016/j.jbi.2008.08.010, PMID: 18929686 PMC2700030

[86] Dubreuil-Vall L Chau P Ruffini G Widge AS Camprodon JA. tDCS to the left DLPFC modulates cognitive and physiological correlates of executive function in a state-dependent manner. Brain Stimul. (2019) 12:1456–63. doi: 10.1016/j.brs.2019.06.006, PMID: 31221553 PMC6851462

[87] Dubreuil-Vall L Gomez-Bernal F Villegas AC Cirillo P Surman C Ruffini G. Transcranial direct current stimulation to the left dorsolateral prefrontal cortex improves cognitive control in patients with attention-deficit/hyperactivity disorder: a randomized behavioral and neurophysiological study. Biol Psychiatry Cogn Neurosci Neuroimaging. (2021) 6:439–48. doi: 10.1016/j.bpsc.2020.11.006, PMID: 33549516 PMC8103824

[88] Rammsayer T Ulrich R. Elaborative rehearsal of nontemporal information interferes with temporal processing of durations in the range of seconds but not milliseconds. Acta Psychol. (2011) 137:127–33. doi: 10.1016/j.actpsy.2011.03.010, PMID: 21474111

[89] Huang Y-Z Sommer M Thickbroom G Hamada M Pascual-Leonne A Paulus W. Consensus: new methodologies for brain stimulation. Brain Stimul. (2009) 2:2–13. doi: 10.1016/j.brs.2008.09.007, PMID: 20633398 PMC5507351

[90] Camprodon JA Pascual-Leone A. Multimodal applications of transcranial magnetic stimulation for circuit-based psychiatry. JAMA Psychiatry. (2016) 73:407–8. doi: 10.1001/jamapsychiatry.2015.3127, PMID: 26981644

[91] Ashida R Cerminara NL Brooks J Apps R. Principles of organization of the human cerebellum: macro- and microanatomy. Handb Clin Neurol. (2018) 154:45–58. doi: 10.1016/B978-0-444-63956-1.00003-5, PMID: 29903451

[92] D'Angelo E. Physiology of the cerebellum. Handb Clin Neurol. (2018) 154:85–108. doi: 10.1016/B978-0-444-63956-1.00006-0 29903454

[93] Silvanto J Muggleton NG Cowey A Walsh V. Neural adaptation reveals state-dependent effects of transcranial magnetic stimulation. Eur J Neurosci. (2007) 25:1874–81. doi: 10.1111/j.1460-9568.2007.05440.x, PMID: 17408427

[94] Wong AL Goldsmith J Forrence AD Haith AM Krakauer JW. Reaction times can reflect habits rather than computations. elife. (2017) 6:6. doi: 10.7554/eLife.28075 PMC5582865 28753125

[95] Andreasen NC Nopoulos P O'Leary DS Miller DD Wassink T Flaum M. Defining the phenotype of schizophrenia: cognitive dysmetria and its neural mechanisms. Biol Psychiatry. (1999) 46:908–20. doi: 10.1016/S0006-3223(99)00152-3, PMID: 10509174

[96] Andreasen NC. A unitary model of schizophrenia. Bleuler's "fragmented Phrene" as schizencephaly. Arch Gen Psychiatry. (1999) 56:781–7. doi: 10.1001/archpsyc.56.9.781 12884883

[97] Kim DJ Kent JS Bolbecker AR Sporns O Cheng H Newman SD. Disrupted modular architecture of cerebellum in schizophrenia: a graph theoretic analysis. Schizophr Bull. (2014) 40:1216–26. doi: 10.1093/schbul/sbu059, PMID: 24782561 PMC4193723

[98] Ding Y Ou Y Pan P Shan X Chen J Liu F. Cerebellar structural and functional abnormalities in first-episode and drug-naive patients with schizophrenia: a meta-analysis. Psychiatry Res Neuroimaging. (2019) 283:24–33. doi: 10.1016/j.pscychresns.2018.11.009, PMID: 30500474

[99] Picard H Amado I Mouchet-Mages S Olie JP Krebs MO. The role of the cerebellum in schizophrenia: an update of clinical, cognitive, and functional evidences. Schizophr Bull. (2008) 34:155–72. doi: 10.1093/schbul/sbm049, PMID: 17562694 PMC2632376

[100] Lungu O Barakat M Laventure S Debas K Proulx S Luck D. The incidence and nature of cerebellar findings in schizophrenia: a quantitative review of fMRI literature. Schizophr Bull. (2013) 39:797–806. doi: 10.1093/schbul/sbr193, PMID: 22267533 PMC3686438

[101] Liang M Zhou Y Jiang T Liu Z Tian L Liu H. Widespread functional disconnectivity in schizophrenia with resting-state functional magnetic resonance imaging. Neuroreport. (2006) 17:209–13. doi: 10.1097/01.wnr.0000198434.06518.b8, PMID: 16407773

[102] Bluhm RL Miller J Lanius RA Osuch EA Boksman K Neufeld RW. Spontaneous low-frequency fluctuations in the BOLD signal in schizophrenic patients: anomalies in the default network. Schizophr Bull. (2007) 33:1004–12. doi: 10.1093/schbul/sbm052, PMID: 17556752 PMC2632312

[103] Shen H Wang L Liu Y Hu D. Discriminative analysis of resting-state functional connectivity patterns of schizophrenia using low dimensional embedding of fMRI. NeuroImage. (2010) 49:3110–21. doi: 10.1016/j.neuroimage.2009.11.011, PMID: 19931396

[104] Liu H Fan G Xu K Wang F. Changes in cerebellar functional connectivity and anatomical connectivity in schizophrenia: a combined resting-state functional MRI and diffusion tensor imaging study. J Magn Reson Imaging. (2011) 34:1430–8. doi: 10.1002/jmri.22784, PMID: 21976249 PMC3221764

[105] Repovs G Csernansky JG Barch DM. Brain network connectivity in individuals with schizophrenia and their siblings. Biol Psychiatry. (2011) 69:967–73. doi: 10.1016/j.biopsych.2010.11.009, PMID: 21193174 PMC3081915

[106] Collin G Hulshoff Pol HE Haijma SV Cahn W Kahn RS van den Heuvel MP. Impaired cerebellar functional connectivity in schizophrenia patients and their healthy siblings. Front Psych. (2011) 2:73. doi: 10.3389/fpsyt.2011.00073 PMC3240868 22203807

[107] Chen YL Tu PC Lee YC Chen YS Li CT Su TP. Resting-state fMRI mapping of cerebellar functional dysconnections involving multiple large-scale networks in patients with schizophrenia. Schizophr Res. (2013) 149:26–34. doi: 10.1016/j.schres.2013.05.029, PMID: 23810119

[108] Guo W Liu F Chen J Wu R Zhang Z Yu M. Resting-state cerebellar-cerebral networks are differently affected in first-episode, drug-naive schizophrenia patients and unaffected siblings. Sci Rep. (2015) 5:17275. doi: 10.1038/srep17275, PMID: 26608842 PMC4660304

[109] Wang H Guo W Liu F Wang G Lyu H Wu R. Patients with first-episode, drug-naive schizophrenia and subjects at ultra-high risk of psychosis shared increased cerebellar-default mode network connectivity at rest. Sci Rep. (2016) 6:26124. doi: 10.1038/srep26124, PMID: 27188233 PMC4870637

[110] Bernard JA Orr JM Mittal VA. Cerebello-thalamo-cortical networks predict positive symptom progression in individuals at ultra-high risk for psychosis. Neuroimage Clin. (2017) 14:622–8. doi: 10.1016/j.nicl.2017.03.001, PMID: 28348953 PMC5357699

[111] Guo W Zhang F Liu F Chen J Wu R Chen DQ. Cerebellar abnormalities in first-episode, drug-naive schizophrenia at rest. Psychiatry Res Neuroimaging. (2018) 276:73–9. doi: 10.1016/j.pscychresns.2018.03.010, PMID: 29628269

[112] Zhuo C Wang C Wang L Guo X Xu Q Liu Y. Altered resting-state functional connectivity of the cerebellum in schizophrenia. Brain Imaging Behav. (2018) 12:383–9. doi: 10.1007/s11682-017-9704-0, PMID: 28293803 PMC5880870

[113] Cao H Chen OY Chung Y Forsyth JK McEwen SC Gee DG. Cerebello-thalamo-cortical hyperconnectivity as a state-independent functional neural signature for psychosis prediction and characterization. Nat Commun. (2018) 9:3836. doi: 10.1038/s41467-018-06350-7, PMID: 30242220 PMC6155100

[114] Ferri J Ford JM Roach BJ Turner JA van Erp TG Voyvodic J. Resting-state thalamic dysconnectivity in schizophrenia and relationships with symptoms. Psychol Med. (2018) 48:2492–9. doi: 10.1017/S003329171800003X, PMID: 29444726 PMC12094034

[115] Lee KH Oh H Suh JS Cho KIK Yoon YB Shin WG. Functional and structural connectivity of the cerebellar nuclei with the striatum and cerebral cortex in first-episode psychosis. J Neuropsychiatry Clin Neurosci. (2018) 31:143–51. doi: 10.1176/appi.neuropsych.17110276 30561280

[116] Breska A Ivry RB. Double dissociation of single-interval and rhythmic temporal prediction in cerebellar degeneration and Parkinson's disease. Proc Natl Acad Sci U S A. (2018) 115:12283–8. doi: 10.1073/pnas.1810596115, PMID: 30425170 PMC6275527

[117] Davalos DB Kisley MA Ross RG. Deficits in auditory and visual temporal perception in schizophrenia. Cogn Neuropsychiatry. (2002) 7:273–82. doi: 10.1080/13546800143000230 16571542

[118] Davalos DB Kisley MA Freedman R. Behavioral and electrophysiological indices of temporal processing dysfunction in schizophrenia. J Neuropsychiatry Clin Neurosci. (2005) 17:517–25. doi: 10.1176/jnp.17.4.517, PMID: 16387992

[119] Carroll CA O'Donnell BF Shekhar A Hetrick WP. Timing dysfunctions in schizophrenia as measured by a repetitive finger tapping task. Brain Cogn. (2009) 71:345–53. doi: 10.1016/j.bandc.2009.06.009, PMID: 19664870 PMC2783288

[120] Elvevag B McCormack T Gilbert A Brown GD Weinberger DR Goldberg TE. Duration judgements in patients with schizophrenia. Psychol Med. (2003) 33:1249–61. doi: 10.1017/S0033291703008122 14580079

[121] Carroll CA O'Donnell BF Shekhar A Hetrick WP. Timing dysfunctions in schizophrenia span from millisecond to several-second durations. Brain Cogn. (2009) 70:181–90. doi: 10.1016/j.bandc.2009.02.001, PMID: 19282082

[122] Allan LG Gibbon J. Human bisection at the geometric mean. Learn Motiv. (1991) 22:39–58. doi: 10.1016/0023-9690(91)90016-2

[123] Rammsayer TH Troche SJ. In search of the internal structure of the processes underlying interval timing in the sub-second and the second range: a confirmatory factor analysis approach. Acta Psychol. (2014) 147:68–74. doi: 10.1016/j.actpsy.2013.05.004, PMID: 23795690

[124] Lewis PA Miall RC. Distinct systems for automatic and cognitively controlled time measurement: evidence from neuroimaging. Curr Opin Neurobiol. (2003) 13:250–5. doi: 10.1016/S0959-4388(03)00036-9, PMID: 12744981

[125] Densen ME. Time perception and schizophrenia. Percept Mot Skills. (1977) 44:436–8. doi: 10.2466/pms.1977.44.2.436 866045

[126] Johnson JE Petzel TP. Temporal orientation and time estimation in chronic schizophrenics. J Clin Psychol. (1971) 27:194–6. doi: 10.1002/1097-4679(197104)27:2<194::AID-JCLP2270270210>3.0.CO;2-F, PMID: 5542463

[127] Tracy JI Monaco C McMichael H Tyson K Chambliss C Christensen HL. Information-processing characteristics of explicit time estimation by patients with schizophrenia and normal controls. Percept Mot Skills. (1998) 86:515–26. doi: 10.2466/pms.1998.86.2.515, PMID: 9638750

[128] Walther S Alexaki D Stegmayer K Vanbellingen T Bohlhalter S. Conceptual disorganization impairs hand gesture performance in schizophrenia. Schizophr Res. (2020) 215:467–8. doi: 10.1016/j.schres.2019.09.001, PMID: 31500999

[129] Huang H Zhang B Mi L Liu M Chang X Luo Y. Reconfiguration of functional dynamics in Cortico-Thalamo-cerebellar circuit in schizophrenia following high-frequency repeated transcranial magnetic stimulation. Front Hum Neurosci. (2022) 16:928315. doi: 10.3389/fnhum.2022.928315, PMID: 35959244 PMC9359206

