# Supplementary material for: Cerebellar transcranial magnetic stimulation in psychotic disorders: intermittent, continuous, and sham theta-burst stimulation on time perception and symptom severity
Source: Front Psychiatry. 2023 Nov 13;14:1218321. doi: 10.3389/fpsyt.2023.1218321 (PMC10679721; doi:10.3389/fpsyt.2023.1218321)

Supplementary Material

Cerebellar transcranial magnetic stimulation in psychotic disorders: intermittent, continuous, and sham theta-burst stimulation on time perception and symptom severity

Ann K. Shinn*, Aura M. Hurtado, Youkyung S. Roh, Victoria Ho, Melissa Hwang, Bruce M. Cohen, Dost Öngür, Joan A. Camprodon

*** Correspondence:** Ann K. Shinn, MD, MPH, akshinn@mgb.org

1 Supplementary Materials about Visual Analog Scales (VAS) for Mood and Psychotic Symptoms

Supplementary Table S1: Mood and Psychotic symptoms

Symptom	Question	Scale labels		
		**VAS rating of 0**	**VAS rating of 50**	**VAS rating of 100**
**Depressed mood**	How depressed do you feel right now?	Not at all depressed	Moderately depressed	The most depressed I have ever felt
**Anxiety**	How anxious, nervous, or worried do you feel right now?	Not at all anxious, nervous, or worried	Moderately anxious, nervous, or worried	The most anxious, nervous, or worried I have ever felt
**Elated mood**	How elated, euphoric, or high do you feel right now?	Not at all elated, euphoric, or high	Moderately elated, euphoric, or high	The most elated, euphoric, or high I have ever felt
**Auditory hallucinations (AH)**	How bothersome are auditory hallucinations (e.g., hearing sounds or voices that others do not seem to hear) right now?	No auditory hallucinations present	Moderately bothersome	Extremely bothersome (the worst I have ever experienced)
**Visual hallucinations (VH)**	How bothersome are visual hallucinations (e.g., seeing things that others do not seem to see) right now?	No visual hallucinations present	Moderately bothersome	Extremely bothersome (the worst I have ever experienced)

**Paranoid ideation (PI)**	Right now, how much do you feel that people are out to get you, bothering you, or watching/monitoring/following you in any way?	Not at all	Possibly so (I question it to some degree)	Definitely so (I am certain of this)
**Ideas/delusions of reference (IOR)**	Right now, how much do you feel that people or things in the environment refer to you, contain special signs or messages for you, or communicate with you in any unusual ways?	Not at all	Possibly so (I question it to some degree)	Definitely so (I am certain of this)
**Delusions of control**	Right now, how much do you feel you are being controlled by some outside force?	Not at all	Possibly so (I question it to some degree)	Definitely so (I am certain of this)

Participants were instructed to indicate self-ratings of each of the above symptoms on a 0-to-100 point visual analog scale (VAS) before and after TMS. Self-ratings were completed via a computerized survey administered in Research Electronic Data Capture (REDCap), which gave instructions to change the position of the slider (originally set to VAS score of 50) to set a response.

Supplementary Table S2. Evaluation of Convergent Validity of VAS Items

VAS item	Standardized Measure	Observations	Spearman's rho	p-value
**Depressed mood**	MADRS total score	26	0.5787	0.002*
	MADRS item 2 (reported sadness)	26	0.4646	0.017*
**Anxiety**	PANSS general psychopathology item G2 (anxiety)	26	0.5227	0.006*
	PANSS general psychopathology item G4 (tension)	26	0.5721	0.002*
**Elated mood**	YMRS total score	26	0.2651	0.191
	YMRS item 1 (elevated mood)	26	0.1573	0.443
**Auditory hallucinations (AH)**	PSYRATS-AH total score	23	0.8035	$3.894 \times 10^{-6*}$
	PANSS positive symptoms item P3 (hallucinations)	26	0.7132	$4.316 \times 10^{-5*}$
	SAPS auditory hallucinations item	25	0.7795	$4.390 \times 10^{-6*}$
**Visual hallucinations (VH)**	PANSS positive symptoms item P3 (hallucinations)	26	0.7519	$9.496 \times 10^{-6*}$

	SAPS visual hallucinations item	25	0.5063	0.010*
**Paranoid ideation (PI)**	PANSS positive symptoms item P6 (suspiciousness)	26	0.5857	0.002*
	PANSS positive symptoms item P1 (delusions)	26	0.5396	0.004*
	SAPS persecutory delusions item	25	0.6646	$3.000 \times 10^{-4*}$
**Ideas/delusions of reference (IOR)**	PANSS positive symptoms item P1 (delusions)	26	0.3926	0.047*
	SAPS ideas and delusions of reference item	25	0.3325	0.104
**Delusions of control**	PANSS positive symptoms item P1 (delusions)	26	0.0137	0.947
	SAPS delusions of being controlled item	25	-0.0493	0.815

Abbreviations (alphabetical): MADRS = Montgomery-Asberg Depression Rating Scale; PANSS = Positive and Negative Syndrome Scale; PSYRATS-AH = Psychotic Symptom Rating Scale, Auditory Hallucinations Subscale; SAPS. = Scale for the Assessment of Positive Symptoms (SAPS); YMRS = Young Mania Rating Scale.

* Asterisk indicates $p < 0.05$.

Supplementary Table S3. Test-Retest Reliability of VAS Items

**Item**	**ICC**	**95% CI**		**Interpretation**[†]
**Depressed mood**	0.4708952	0.2353944	0.7201077	Fair
**Anxiety**	0.48284	0.2466258	0.7269804	Fair
**Elated mood**	0.3345128	0.1221445	0.6448755	Slight
**Auditory hallucinations (AH)**	0.8421572	0.7013688	0.9237842	Substantial
**Visual hallucinations (VH)**	0.6699219	0.4523201	0.8329895	Moderate
**Paranoid ideation (PI)**	0.6415167	0.4172204	0.817291	Moderate
**Ideas/delusions of reference (IOR)**	0.5635832	0.3282919	0.7733554	Fair
**Delusions of control**	0.2663733	0.0762521	0.6149555	Slight

[†] ICC′s were interpreted using the following guidelines: virtually no (0.00–0.10), slight (0.11–0.40), fair (0.41–0.60), moderate (0.61–0.80), or substantial (0.81–1.0) reliability (Shrout 1998).

Shrout, P.E., 1998. ′Measurement reliability and agreement in psychiatry. Stat. Methods Med. Res. 7, 301–17.

2 Supplementary Data for Completers (N=20)

Supplementary Figure S1. Spaghetti Plots of Within-Subject Interval Discrimination Task (IDT) Reaction Time Changes Pre- and Post-TBS (for Completers Only, n=20)

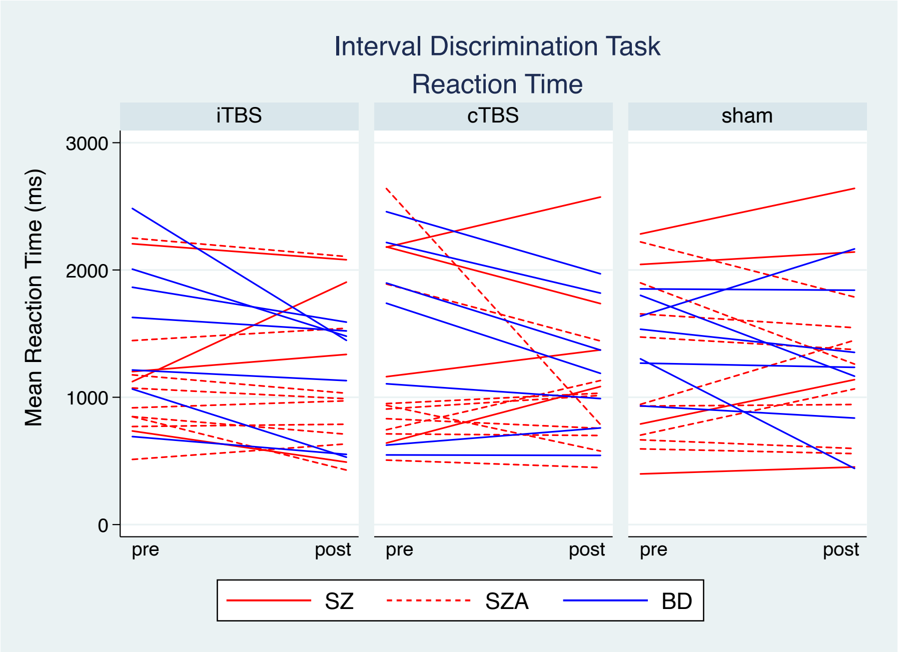

Supplementary Table S4. Changes in mean Interval Discrimination Task (IDT) accuracy pre- and post-TBS (for Completers Only, n=20†)

**IDT Accuracy††**	**All Conditions**	**iTBS**	**cTBS**	**Sham**
No. of participants	n=20	n=20	n=20	n=20
Pre-TBS	0.497 ± 0.166 (0.133 to 0.867)†††	0.503 ± 0.161 (0.133 to 0.733)	0.523 ± 0.188 (0.267 to 0.867)	0.463 ± 0.151 (0.267 to 0.733)
Post-TBS	0.509 ± 0.150 (0.267 to 0.867)†††	0.493 ± 0.125 (0.267 to 0.800)	0.513 ± 0.148 (0.267 to 0.800)	0.520 ± 0.178 (0.267 to 0.867)
$\Delta_{\text{post-pre}}$	0.012 ± 0.182 (-0.333 to 0.467)†††	-0.010 ± 0.175 (-0.333 to 0.333)	-0.010 ± 0.167 (-0.333 to 0.200)	0.057 ± 0.204 (-0.200 to 0.467)

† Results presented for only the subset of n=20 participants who completed all three study visits (iTBS, cTBS, and sham).
†† Accuracy calculated as the proportion of interval discrimination task (IDT) trials performed correctly. Data reported as mean ± SD (range).
††† Means ± SD (ranges) for all three conditions (iTBS, cTBS, sham) across the 20 completers includes data from 60 different sessions (3 observations for each participant).

Supplementary Figure S2. Spaghetti Plots of Within-Subject Interval Discrimination Task (IDT) Accuracy Changes Pre- and Post-TBS (for Completers Only, n=20)

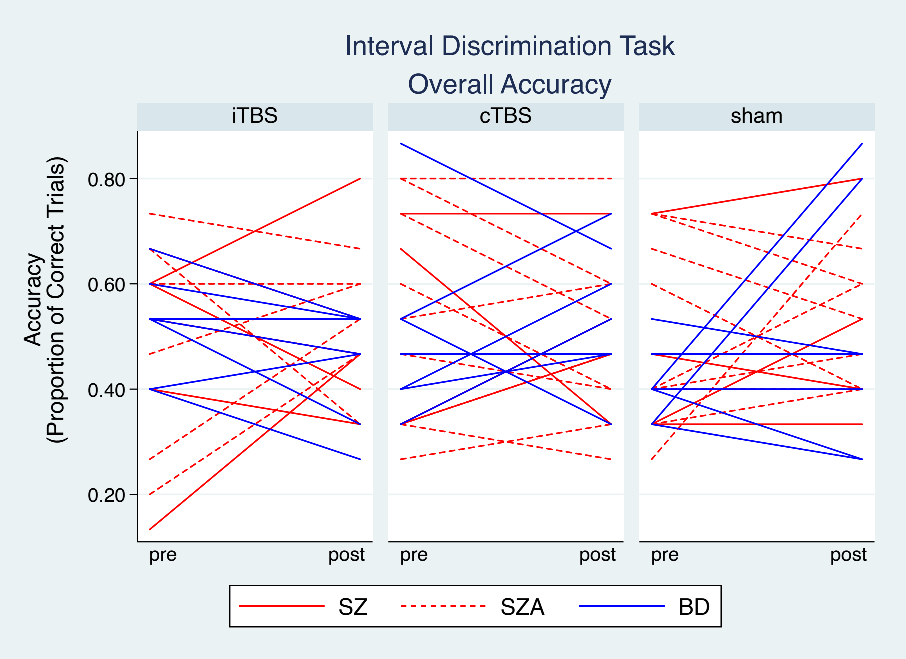

Supplementary Table S5. Mixed effects model for Interval Discrimination Task (IDT) Accuracy (for Completers Only, n=20)

	**Coefficient**	**Std error**	**Test statistic**	***P*-value**	**95% CI**
**Post-TMS (vs. pre)**	0.05667	0.03985	$z = 1.42$	$p = 0.155$	-0.02143 to 0.13476
**iTBS (vs. sham)**	0.04000	0.03985	$z = 1.00$	$p = 0.315$	-0.03810 to 0.11810
**cTBS (vs. sham)**	0.06000	0.03985	$z = 1.51$	$p = 0.132$	-0.01810 to 0.13810
**Post-TMS x iTBS**	-0.06667	0.05635	$z = -1.18$	$p = 0.237$	-0.17711 to 0.04378
**Post-TMS x cTBS**	-0.06667	0.05635	$z = -1.18$	$p = 0.237$	-0.17711 to 0.04378
**Intercept**	0.46333	0.03572	$z = 12.97$	$p < 0.0001$	0.39332 to 0.53335

The mixed effects regression model included time (pre, post), condition (iTBS vs. sham, cTBS vs. sham), and time x condition interaction terms as independent variables, and interval discrimination task (IDT) performance accuracy as the dependent variable. The primary hypotheses in the analysis relate to the time x iTBS and time x cTBS interaction terms, neither of which meet the significance threshold of $p < 0.05$, two-sided, uncorrected.

Supplementary Table S6. Changes in mood and psychotic symptoms pre- vs. post-TMS within each TMS condition (for Completers Only, n=20[†])

Symptom	Time	Visual Analog Scale (VAS) Self-Rating[††]			Test statistic	*p*-value[†††]
		**iTBS**	**cTBS**	**Sham**		
**Depressed mood**	Pre-TBS	25.5 (0 to 70)	37 (0 to 91)	15 (0 to 70)		
	Post-TBS	28.5 (0 to 72)	42 (0 to 71)	7 (0 to 76)		
	$\Delta_{post\text{-}pre}$	0 (-31 to 63)	0 (-41 to 26)	0 (-43 to 7)	Q = 0.197	*p* = 0.906
**Anxiety**	Pre-TBS	20.5 (0 to 73)	50 (0 to 87)	22 (0 to 74)		
	Post-TBS	2.5 (0 to 75)	36 (0 to 77)	4.5 (0 to 73)		
	$\Delta_{post\text{-}pre}$	-2.5 (-50 to 24)	-1 (-40 to 32)	0 (-30 to 22)	Q = 0.394	*p* = 0.821
**Auditory hallucinations**	Pre-TBS	0 (0 to 65)	0 (0 to 50)	0 (0 to 48)		
	Post-TBS	0 (0 to 49)	0 (0 to 50)	0 (0 to 65)		
	$\Delta_{post\text{-}pre}$	0 (-44 to 1)	0 (-9 to 48)	0 (-9 to 50)	Q = 5.879	*p* = 0.053
**Visual hallucinations**	Pre-TBS	0 (0 to 52)	0 (0 to 79)	0 (0 to 50)		
	Post-TBS	0 (0 to 53)	0 (0 to 56)	0 (0 to 50)		
	$\Delta_{post\text{-}pre}$	0 (-22 to 9)	0 (-29 to 8)	0 (-13 to 50)	Q = 0.146	*p* = 0.929
**Paranoid ideation**	Pre-TBS	0.5 (0 to 55)	0 (0 to 63)	0 (0 to 71)		
	Post-TBS	0 (0 to 50)	0 (0 to 59)	0 (0 to 100)		
	$\Delta_{post\text{-}pre}$	0 (-24 to 41)	0 (-43 to 3)	0 (-18 to 43)	Q = 6.745	*p* = **0.034**[1]

† Results presented for only the subset of n=20 participants who completed all three study visits (iTBS, cTBS, and sham).

†† Medians (ranges) reported, as data are not normally distributed.

†† *p*-values derived by conducting Friedman's non-parametric repeated measures ANOVA to test the null hypothesis that there is no effect of condition (iTBS, cTBS, sham) on $\Delta_{post\text{-}pre}$ in VAS symptom self-ratings. *P*-values in bold font meet the significance threshold of $p < 0.05$, two-sided, uncorrected. No findings survive Bonferroni correction.

[1] Post-hoc pairwise testing with Wilcoxon signed rank tests (threshold $p < 0.05$, uncorrected) indicate a significant difference between cTBS and sham.

Supplementary Figure S3. Changes (Δ $_{post-pre}$) in mood and psychotic symptom self-ratings before and after intermittent, continuous, and sham theta burst stimulation (TBS) (for Completers Only, n=20)

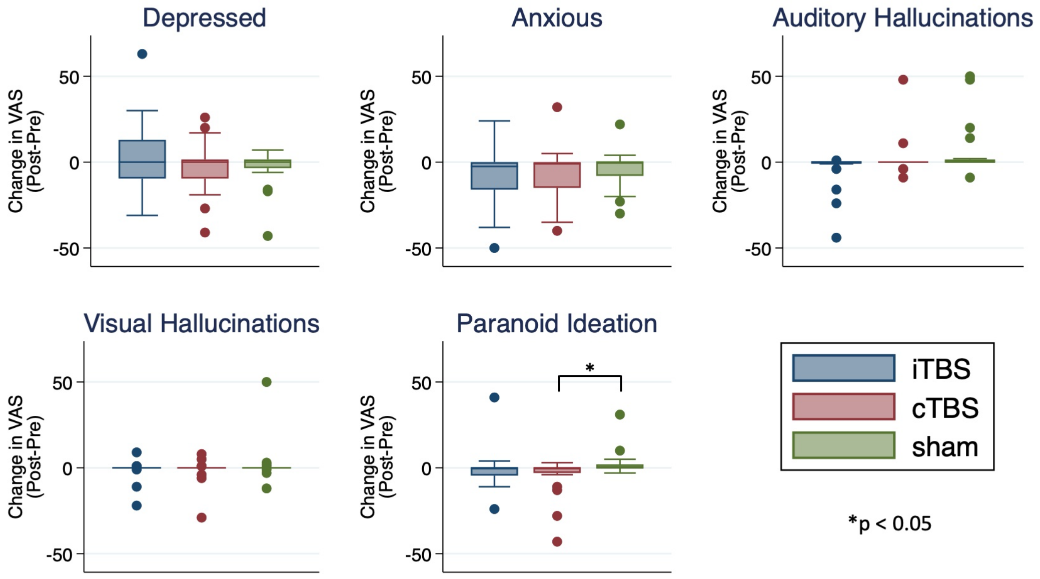

Box and whisker plots showing changes (Δ $_{post-pre}$) in visual analog scale (VAS) symptom self-ratings before and after administration of a single session of intermittent (iTBS), continuous (cTBS), or sham theta burst stimulation (TBS). Here, data are shown for only the subset of n=20 participants who completed all three study visits (iTBS, cTBS, and sham). The TBS conditions differed significantly in their effects on self-ratings of paranoid ideation (PI). Post-hoc pairwise comparisons showed that the significant difference was between cTBS and sham (z=2.227, p=0.026) for PI. See Supplementary Figure S3 for visualization of changes in VAS symptom self-ratings for all participants (n=26).

Supplementary Figure S4. Spaghetti Plots of Within-Subject Changes in Symptom Self-Ratings Pre- and Post-TBS (for Completers Only, n=20)

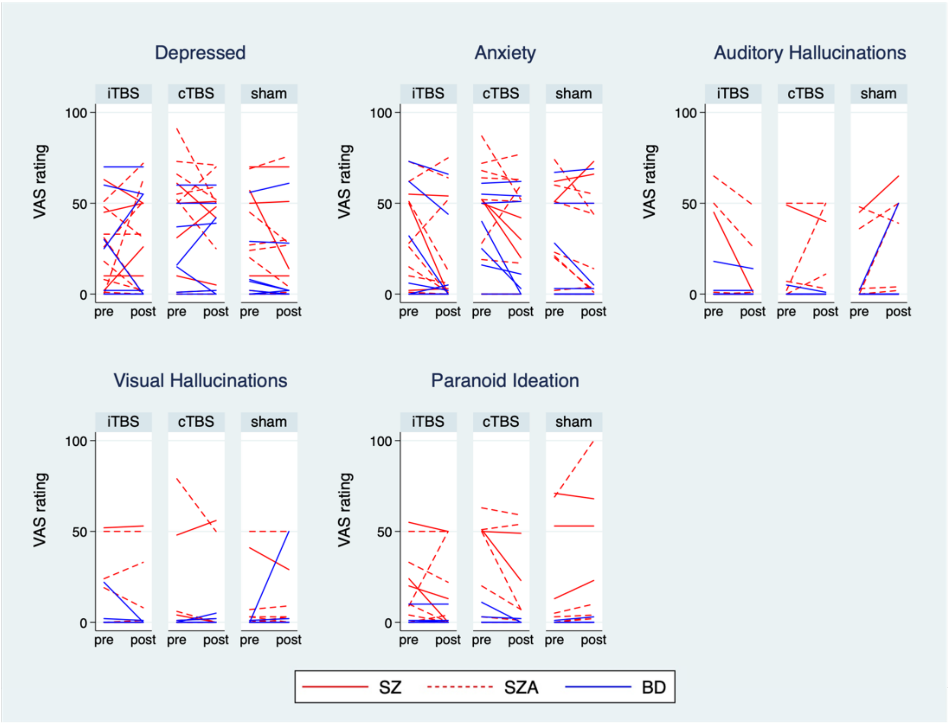

3 Supplementary Data for All Participants (n=26)

Supplementary Figure S5. Changes in Interval Discrimination Task (IDT) reaction time pre- and post-TBS (for All Participants, n=26)

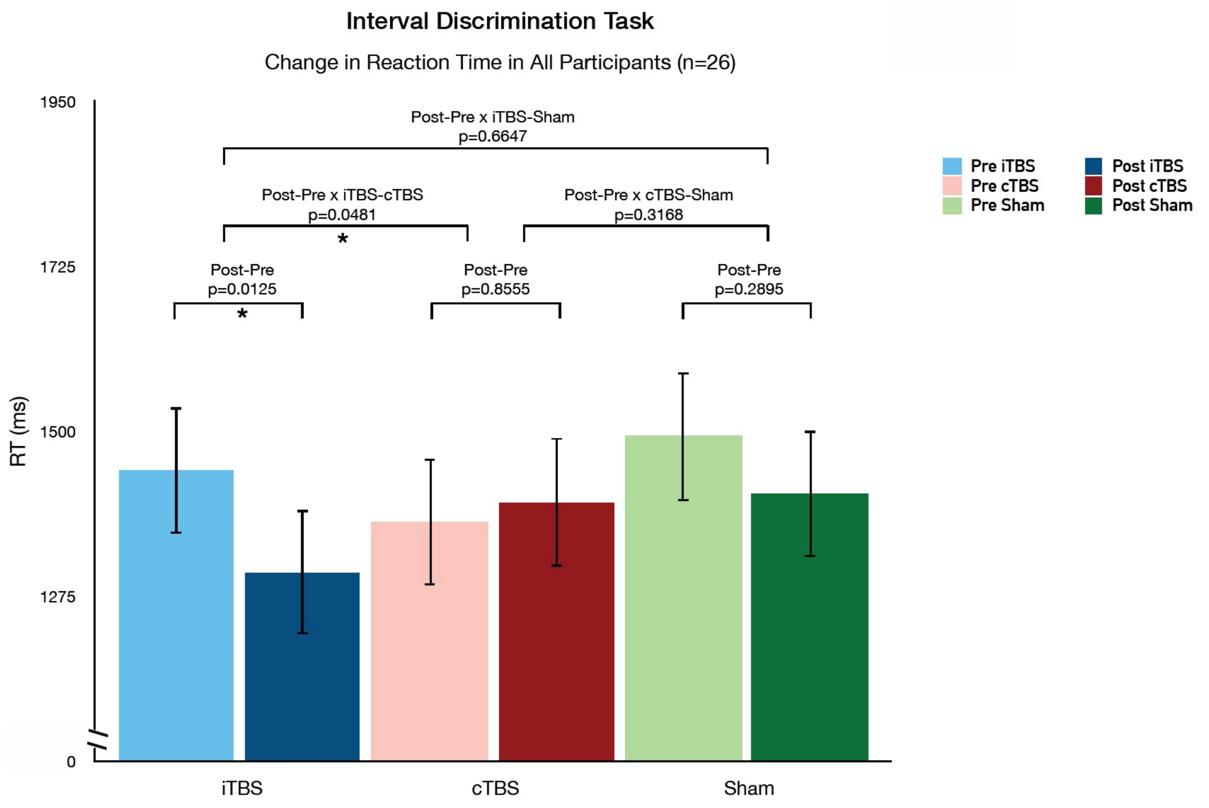

Supplementary Table S7. Least square (LS) means for pre- and post-TBS conditions (for All Participants, n=26)

TBS Condition	Time	LS-mean	Standard Error
**iTBS**	**Pre**	1446 ms	87.1
	**Post**	1307 ms	85.2
**cTBS**	**Pre**	1376 ms	86.6
	**Post**	1403 ms	86.9
**Sham**	**Pre**	1493 ms	87.1
	**Post**	1415 ms	85.9

Supplementary Figure S6. Spaghetti Plots of Within-Subject Interval Discrimination Task (IDT) Reaction Time Changes Pre- and Post-TBS (for All Participants, n=26)

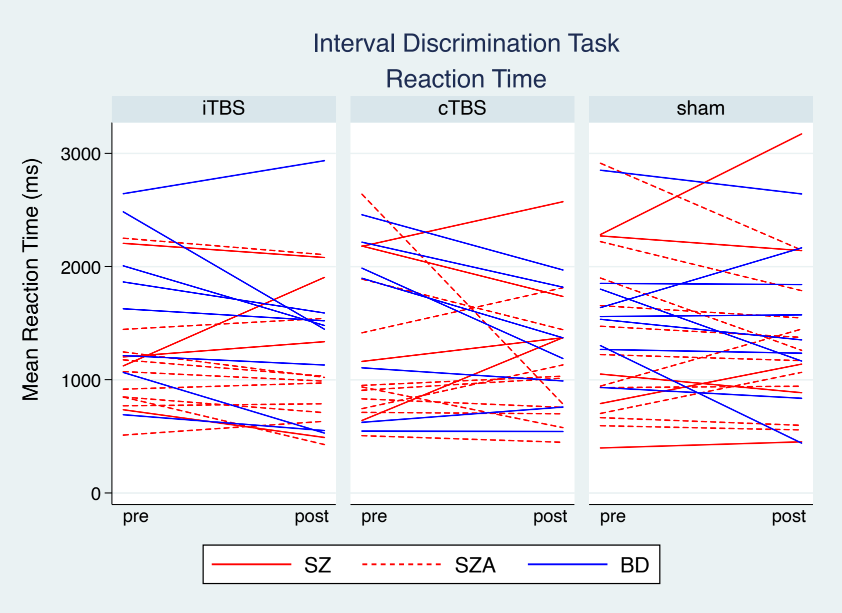

Supplementary Table S8. Changes in mean Interval Discrimination Task (IDT) accuracy pre- and post-TBS (for All Participants, n=26†)

**IDT Accuracy††**	**All Conditions**	**iTBS**	**cTBS**	**Sham**
No. of participants	n=26	n=22	n=21	n=25
Pre-TBS	0.491 ± 0.160<br>(0.133 to 0.867)†††	0.503 ± 0.156<br>(0.133 to 0.733)	0.521 ± 0.183<br>(0.267 to 0.867)	0.456 ± 0.141<br>(0.267 to 0.733)
Post-TBS	0.507 ± 0.150<br>(0.267 to 0.867)†††	0.479 ± 0.128<br>(0.267 to 0.800)	0.514 ± 0.145<br>(0.267 to 0.800)	0.525 ± 0.174<br>(0.267 to 0.867)
$\Delta_{\text{post-pre}}$	0.015 ± 0.183<br>(-0.333 to 0.467)†††	-0.024 ± 0.175<br>(-0.333 to 0.333)	-0.006 ± 0.163<br>(-0.333 to 0.200)	0.069 ± 0.198<br>(-0.200 to 0.467)

† Results presented for all participants with data (i.e., all participants who completed at least one study visit).
†† Accuracy calculated as the proportion of interval discrimination task (IDT) trials performed correctly. Data reported as mean ± SD (range).
††† Means ± SD (ranges) for all three conditions (iTBS, cTBS, sham) across the 26 participants include data from 68 different sessions (1-3 observations for each participant).

Supplementary Table S9. Mixed effects model for Interval Discrimination Task (IDT) Accuracy (for All Participants, n=26)

	**Coefficient**	**Std error**	**Test statistic**	***P*-value**	**95% CI**
**Post-TMS (vs. pre)**	0.06933	0.03493	$z = 1.99$	$p = 0.047$	0.00089 to 0.13779
**iTBS (vs. sham)**	0.05747	0.03588	$z = 1.60$	$p = 0.109$	-0.01285 to 0.12778
**cTBS (vs. sham)**	0.06478	0.03711	$z = 1.75$	$p = 0.081$	-0.00795 to 0.13751
**Post-TMS x iTBS interaction**	-0.09934	0.05082	$z = -1.95$	$p = 0.051$	-0.19894 to 0.00026
**Post-TMS x cTBS interaction**	-0.07569	0.05169	$z = -1.46$	$p = 0.143$	-0.17699 to 0.02563
**Intercept**	0.45563	0.03097	$z = 14.71$	$p < 0.0001$	0.39492 to 0.51634

The mixed effects regression model included time (pre, post), condition (iTBS vs. sham, cTBS vs. sham), and time x condition interaction terms as independent variables, and interval discrimination task (IDT) performance accuracy as the dependent variable. The primary hypotheses in the analysis relate to the time x iTBS and time x cTBS interaction terms, neither of which meet the significance threshold of $p < 0.05$, two-sided, uncorrected.

Supplementary Figure S7. Spaghetti Plots of Within-Subject Interval Discrimination Task (IDT) Accuracy Changes Pre- and Post-TBS (for All Participants, n=26)

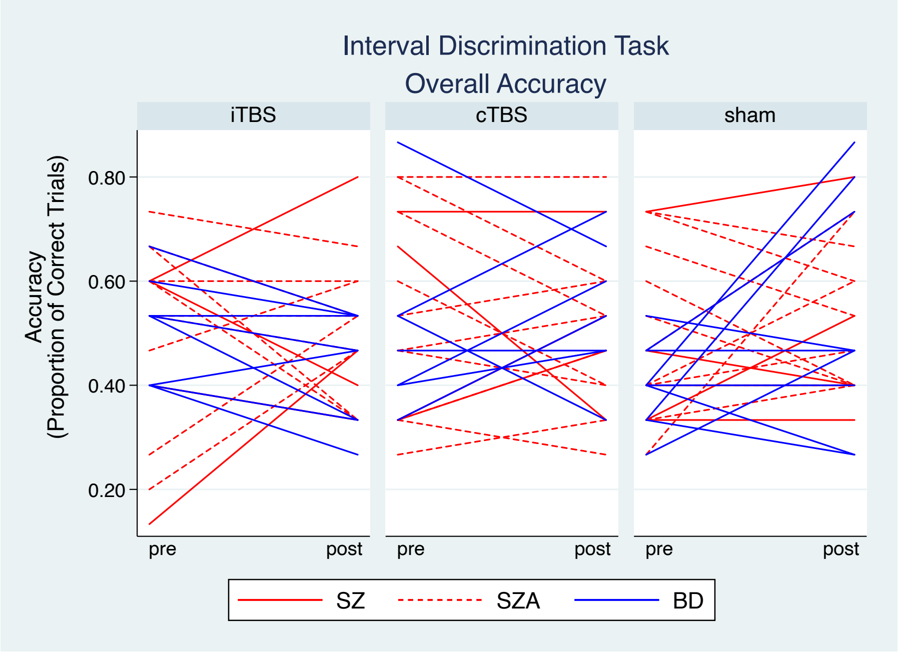

Supplementary Table S10. Changes in symptoms pre- vs. post-TMS within each TMS condition (for All Participants, n=26)

**Symptom**	**iTBS† Δ $_{post-pre}$**	**cTBS† Δ $_{post-pre}$**	**Sham† Δ $_{post-pre}$**	**Test Statistic**	***p*-value††**
**Depressed mood**	0 (-31 to 63)	0 (-41 to 26)	0 (-43 to 7)	Q = 0.188	*p* = 0.910
**Anxiety**	-2.5 (-50 to 24)	-1 (-40 to 32)	0 (-30 to 22)	Q = 0.373	*p* = 0.830
**Auditory hallucinations**	0 (-44 to 1)	0 (-9 to 48)	0 (-9 to 50)	Q = 6.709	*p* = **0.035**
**Visual hallucinations**	0 (-22 to 9)	0 (-29 to 8)	0 (-13 to 50)	Q = 0.581	*p* = 0.748
**Paranoid ideation**	0 (-24 to 41)	0 (-43 to 3)	0 (-18 to 43)	Q = 7.872	*p* = **0.020**

Medians (ranges) reported

† n=22 of the 26 participants completed the iTBS study visit; n=21 completed cTBS; n=25 completed sham.

†† *p*-values derived by conducting Friedman's non-parametric repeated measures ANOVA to test the null hypothesis that there is no effect of condition (iTBS, cTBS, sham) on Δ $_{post-pre}$ in VAS symptom scores. *P*-values in bold font meet the significance threshold of $p < 0.05$, two-sided, uncorrected. No findings survive Bonferroni correction.

Supplementary Figure S8. Changes (Δ $_{post-pre}$) in mood and psychotic symptom self-ratings before and after intermittent, continuous, and sham theta burst stimulation (TBS) (for All Participants, n=26)

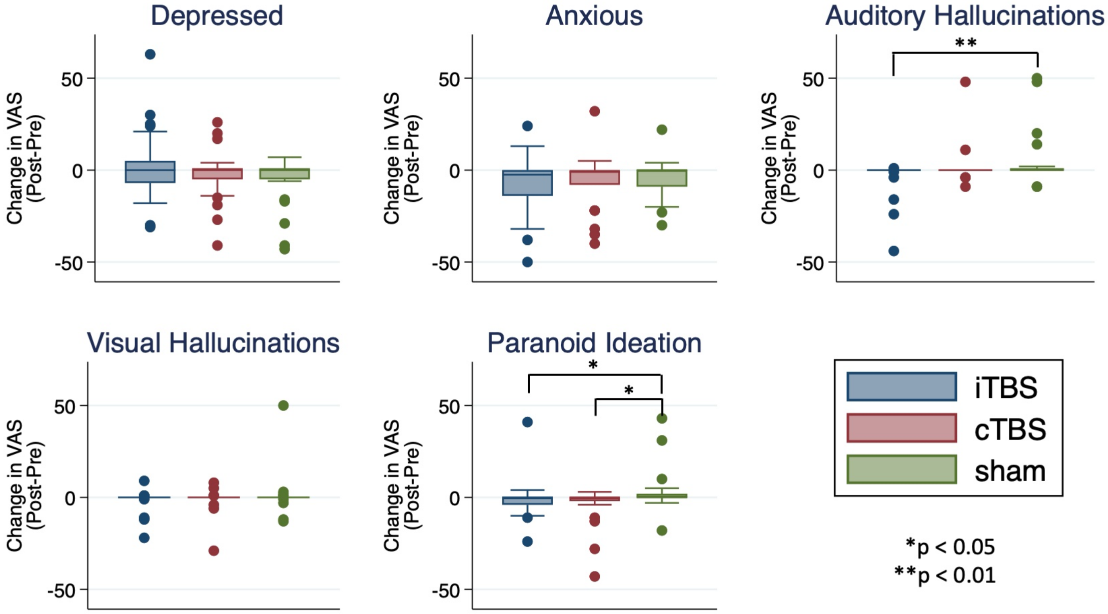

Box and whisker plots showing changes (Δ $_{post-pre}$) in participants' (n=26) visual analog scale (VAS) symptom self-ratings before and after administration of a single session of intermittent (iTBS), continuous (cTBS), or sham theta burst stimulation (TBS). The TBS conditions differed significantly in their effects on self-ratings of auditory hallucinations (AH) and paranoid ideation (PI). Post-hoc pairwise comparisons showed that the significant difference was between iTBS and sham (z=2.639, p=0.008) for AH, and between iTBS and sham (z=2.283, p=0.022) as well as between cTBS and sham (z=2.227, p=0.026) for PI. See Supplementary Figure S1 for visualization of changes in VAS symptom self-ratings for only the completers (n=20).

Supplementary Figure S9. Spaghetti plots of within-subject changes in symptom self-ratings before and after intermittent, continuous, and sham theta burst stimulation (TBS) (for All Participants, n=26)

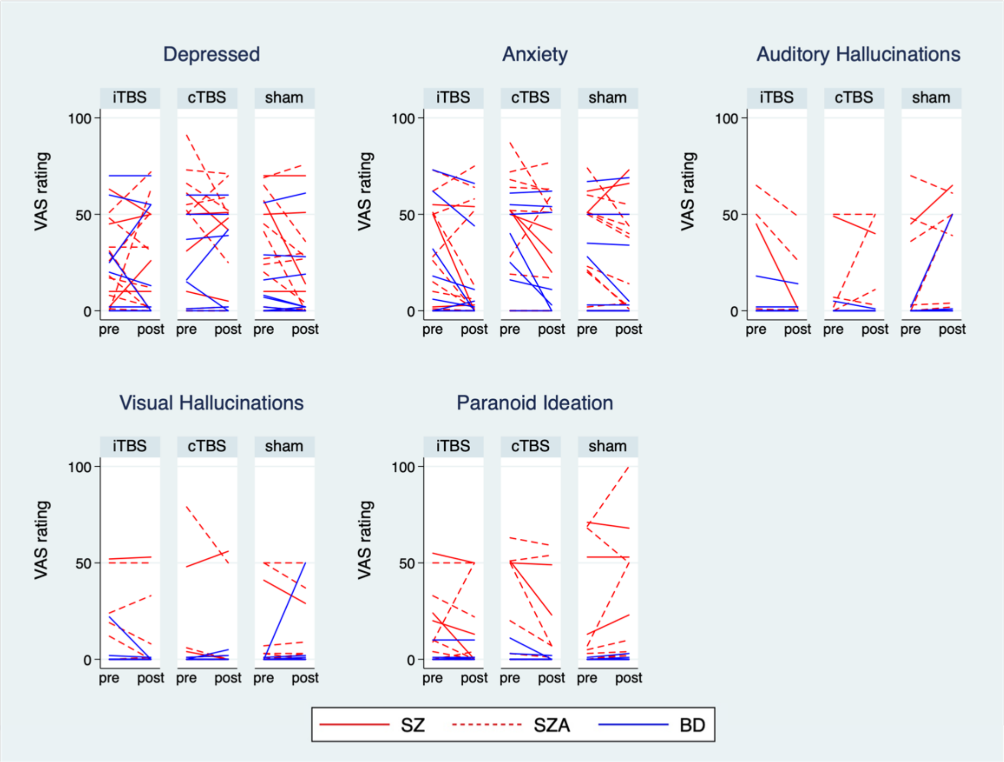

Supplement: Supplementary file 1 [file Data_Sheet_1.pdf]
